# Prophage Activation Triggers Extracellular Synthesis of DNA in Bacterial Biofilms via Rolling Circle Replication

**DOI:** 10.1002/anie.3695434

**Published:** 2026-08-24

**Authors:** Gabriel Antonio S. Minero, Line Mørkholt Lund, Lasse Hyldgaard Klausen, Obinna Markraphael Ajunwa, Mingdong Dong, Rikke Louise Meyer, Victoria Birkedal, Kai Thormann

**Affiliations:** ^1^ Interdisciplinary Nanoscience Center (iNANO) Aarhus University Aarhus Denmark; ^2^ Department of Biology Aarhus University Aarhus Denmark; ^3^ Department of Chemistry Aarhus University Aarhus Denmark; ^4^ Center of Electromicrobiology (CEM) Department of Biology Aarhus University Aarhus Denmark; ^5^ Institut für Mikrobiologie und Molekularbiologie Justus‐Liebig‐Universität Gießen Gießen Germany

**Keywords:** *Bacillus subtilis*, bacterial lysis control, biofilm engineering, DNA replication, extracellular DNA synthesis, G‐quadruplex deoxyribozyme, molecular machines, prophage, rolling circle replication, *Shewanella oneidensis*

## Abstract

Extracellular DNA (eDNA) plays crucial roles in biofilm formation and function, yet the role of bacteriophages, which can exist in a dormant state as prophages, in controlling eDNA synthesis, structure, and activity remains obscure. Here, we demonstrate that prophages harbored by environmental bacteria can be exploited for synthesis of programmable eDNA superstructures with a distinct function. We designed a custom circular template and used it to direct rolling circle replication of G‐quadruplex (GQ) motifs in *Shewanella oneidensis* and *Bacillus subtilis*. Under nutrient‐limiting conditions, prophage activation and proliferation triggered cell lysis and subsequent eDNA synthesis, producing multimeric GQ concatemers that self‐assembled into spherical (≤10 µm) and wire‐like (>50 µm) superstructures in *S. oneidensis* and *B. subtilis*, respectively. Real‐time monitoring using fluorescent reporter strains revealed that DNA synthesis occurred predominantly after bacterial lysis, coinciding with prophage replication. The resulting eDNA superstructures enhanced the electrochemical properties of *S. oneidensis* and exhibited peroxidase activity through GQ‐hemin DNAzyme formation in *S. oneidensis* and *B. subtilis* cocultured biofilm. This work unveils a previously unknown mechanism by which prophages contribute to biofilm architecture and establishes a novel biotechnological platform for engineering functional DNA materials in living bacterial communities with potential applications in synthetic biology.

## Graphical abstract

*Shewanella oneidensis* and *Bacillus subtilis*, both harboring prophages, reveal a novel biosynthetic route to scalable and programmable DNA superstructures in flower‐ and wire‐like shapes, respectively. Activation of prophages is achieved via 3‐hour starvation and monitored using reporter strains. Synthesis of functional GQ DNA from an exogeneous circular template is monitored via specific fluorescent probes.

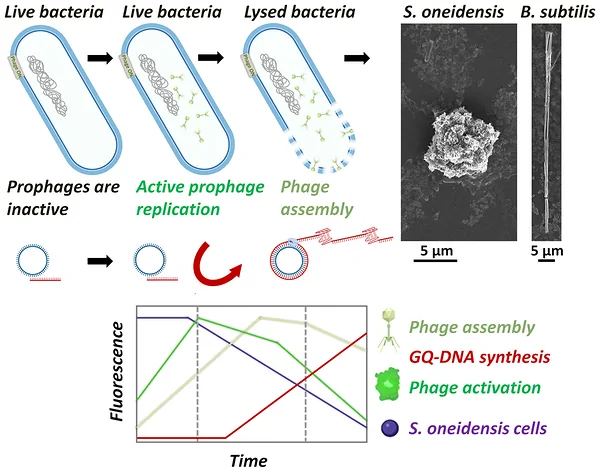

## 1 Introduction

Bacteria release and harness nucleic acids as structural materials in biofilms—microbial communities encased in self‐produced polymeric matrices [1]. While extracellular DNA (eDNA) is known to provide structural support for the biofilm's extracellular matrix, recent research also points to eDNA as a catalyst and a conduit for extracellular electron transfer (EET). The EET is facilitated by eDNA's ability to bind redox‐active molecules like phenazines [2] or hemin [3], allowing bacteria to respire in the absence of electron acceptors by ferrying electrons to an electrode or to oxygen located at some distance from the cell [4]. The unique structural properties of noncanonical DNA forms play a key role in eDNA‐mediated EET [3]. G‐quadruplexes (GQ) were shown to scaffold bacterial redox‐active molecules such as flavins [5] and cytochromes, maintaining them in spatial arrangement for a rapid electron transfer [6]. GQs facilitate π‐π interactions and bind hemin with high specificity, creating a GQ/hemin DNAzyme with peroxidase‐like activity [7]. The DNAzyme activity provides an additional mode for EET from bacterial metabolic processes to hydrogen peroxide and potentially other electron acceptors, which could catalyze CO_2_ reduction [8, 9] or degrade micropollutants [10]. EET mediated by GQ/hemin may therefore augment microbial processes in biofilms used in for example *Bacillus subtilis* (*B. subtilis*) or *Shewanella oneidensis* (*S. oneidensis*) microbial fuel cells [11] or activated sludge [12] to tackle critical environmental challenges. Inducing catalytic DNA production to augment these processes requires knowledge about how they form, and their formation can be stimulated in the complex microbial communities that carry out these processes.

In addition to bacteria, biofilms also contain bacterial viruses (bacteriophages or phages). These phages can facilitate eDNA release from cells into the biofilm's extracellular matrix [13, 14, 15]. Globally, phages outnumber bacteria tenfold [16, 17]. Virulent phages infect and replicate inside bacterial cells, causing cell lysis and the extracellular release of up to hundreds of new phage virions [18]. In contrast, temperate phages can lie dormant in bacterial genomes as prophages, which are activated by certain conditions that trigger the SOS response to oxidative stress and DNA damage, for example in the presence of mitomycin C [18, 19] or iron [13]. Prophages are present across a variety of bacterial species [17, 20] and the mechanisms for prophage activation vary [21]. The SOS response can also be induced in cells post‐starvation, that is upon reintroduction of carbon [22]. Therefore, starvation‐induced SOS response can be utilized as a generic approach to activate some prophages in environmental species and to stimulate eDNA release along with progeny phages.

Some phages, including prophages λSo/ MuSo in *S. oneidensis* and SPP/ SPβ in *B. subtilis*, encode unique DNA polymerases that produce long single‐stranded DNA (ssDNA) from circular ssDNA templates by rolling circle replication (RCR) [23, 24, 25]. Phage polymerases can thus be used to synthesize large quantities of multimeric DNA cocoons in vitro from circular templates [26]. When not limited by nucleoside triphosphates (NTPs), circular DNA replication can produce linear concatemer products made of thousands of “stitched” complementary copies of the circular DNA template. These products can even be programmed to include multimeric GQ‐DNA [27]. Furthermore, the incorporation of NTPs into growing DNA chains releases pyrophosphate (PP) anions as PP precipitates. These insoluble PPs can then nucleate the formation and phase separation of micrometer‐sized flower‐like DNA superstructures in cell‐free systems [28, 29]. The size and morphology of these “DNA flowers” is unique and depends on the sequence and concentration of the DNA template, the type of divalent cation and even the type of the polymerase [30]. DNA sequence, structure, and supramolecular organization are therefore all intimately connected. While this phenomenon has been demonstrated in vitro, it has never been reproduced in bacteria or their biofilms.

*B. subtilis* and *S. oneidensis* are two environmental strains that are being harnessed for applications such as wastewater treatment, CO_2_ capture, and bioenergy production [31, 32]. We hypothesized that upon cell lysis these strains release phage polymerases and NTPs that can continue to synthesize DNA in biofilm matrix in the crowded microenvironment around lysed bacteria. Subsequently, sequence‐specific DNA structures and topologies can be synthesized and consequently integrated into bacterial eDNA matrices by promoting eDNA replication using the desired exogeneous template. Considering the structural importance of eDNA and the other key eDNA functions, for example, EET [3], incorporating specific DNA motifs into the biofilm matrix can enhance biofilm's form and function. The aim of this study was therefore to explore synthesis, shapes, and functions of eDNA by template‐directed synthesis of functional DNA structures in the extracellular matrix of biofilms from the environmental microorganisms *S. oneidensis* and *B. subtilis*. The outcomes of this study will enable enhanced performance of the key environmental bioprocesses using, for example, electroactive *S. oneidensis* microbial fuel cells [11] and bioenergy production [31, 32].

## 2 Results

### 2.1 eDNA Synthesis Is Mediated by Phage Activation and Proliferation

We first sought to determine if eDNA replication can occur in bacterial cultures where NTPs, polymerases and other cellular materials are released by phage‐induced cell lysis. To test this hypothesis, we added a circular DNA template to bacterial cultures, which would produce a detectable product if replicated by phage polymerases capable of rolling circle replication. Assuming that the template is only available extracellularly, the measured product would indicate eDNA replication.

We used a circular template T1 (Table 1) generating a concatemer comprised of several hundred GQ motifs (GQ‐DNA), that may form stacked G‐tetrads stabilized by Hoogsteen hydrogen bonds between the guanines and that, furthermore, may be repurposed for environmental applications [33]. This template was chosen as GQ motifs are detectable using fluorescent probes [34, 35, 36]. To detect RCR in real time (Figures 1 and S1), we used GQ‐specific N‐Methyl Protoporphyrin (NMP) dye [36]. eDNA synthesis by RCR from T1 (Figure 1A) was indicated by an increase in the NMP difference (minus the negative control NMP fluorescence in the non‐templated cultures). GQ‐DNA synthesis by *S. oneidensis* S2391 (*tn7*::CFP, *cro*::Venus) (Figure 1B and C, red) and S5558 (ΔMuSoI ΔMuSoII; Lambda SO_2963 fused to sfGFP) (Figure 1D, red) cultures commenced after a 300 min lag phase. At the same time, cyan fluorescent protein (CFP), representing living intact bacteria, decreased over time starting from about 200 min (Figure 1B and C, violet), indicating that bacteria were lysing due to prophage induction (hereafter referred to as phage activation). The Venus fluorescence (indicative of phage activation) peaked around 300 min (Figure 1B–C, green) and the signal of superfolder green fluorescent protein (sfGFP) fused to λSo capsid (indicative of phage assembly) peaked at 400 min (Figure 1D, yellow‐green). GQ‐DNA therefore accumulated in response to phage proliferation and lysis of the *S. oneidensis* wild‐type and ΔMuSoI ΔMuSoII mutant. Phage λSo polymerase was ultimately responsible for GQ‐DNA replication (Figure 1: red curves). On the contrary, there was no increase in NMP difference in the S6610 (ΔλSo ΔMuSoI ΔMuSoII) strain lacking any prophages (Figure 1B–C: blue curves). The timing of GQ‐DNA synthesis (starting around 300 min in the starved cultures) coincided with activation of prophages detected in the reporter strains of *S. oneidensis* (Figure 1B–C green and red curves indicative of prophage activation and GQ synthesis, respectively; Figure 1D yellow‐green curve indicative of phage λSo capsid production), supporting our hypothesis that T1‐derived RCR occurred extracellularly following phage‐induced lysis of *S. oneidensis*.

**TABLE 1:** The oligonucleotides purchased from IDT and used for the synthetic ligation of circular DNA. Underlined are complementary regions between DNA “Universal primer” and “Templates.” In bold are the motifs “encoding” for GQ‐DNA synthesis. The circularizing oligonucleotides were phosphorylated at the 5’‐end. A complementary DNA oligonucleotide (cDNA) contained 3’‐dideoxycytosine and 5’‐Alexa647.

**Universal primer**	5’ CGGTAAGGACGCGATCACCA CCGGCATCGAGGTCGTATG
Universal primer*	**Alexa405**‐5’ CGGTAAGGACGCGATCACCA CCGGCATCGAGGTCGTATG
**(GQ) T1‐ template**	**P**‐5’ TGGTGATCGCGTCCTTACCGA**CCCACCCTACCCACCC**TCAAATAGTGCAGTATACTTGACT GACCTGCCACAAA**CCCACCCTACCCACCC**TCACATACGACCTCGATGCCGG
**(R) T2‐template**	**P**‐5’ TGGTGATCGCGTCCTTACCGAGTAGCCTTCCCGAGCATTAGTGCAGTATACTTGACT GACCTGCCACGTAATAGTGTCTTACATACGACCTCGATGCCGG
**cDNA**	5’ AGTGCAGTATACTTGACTAAAAAAT ddC **Alexa647**‐5’ AGTCAAGTATACTGCACTAAAAAAT ddC

**FIGURE 1:**
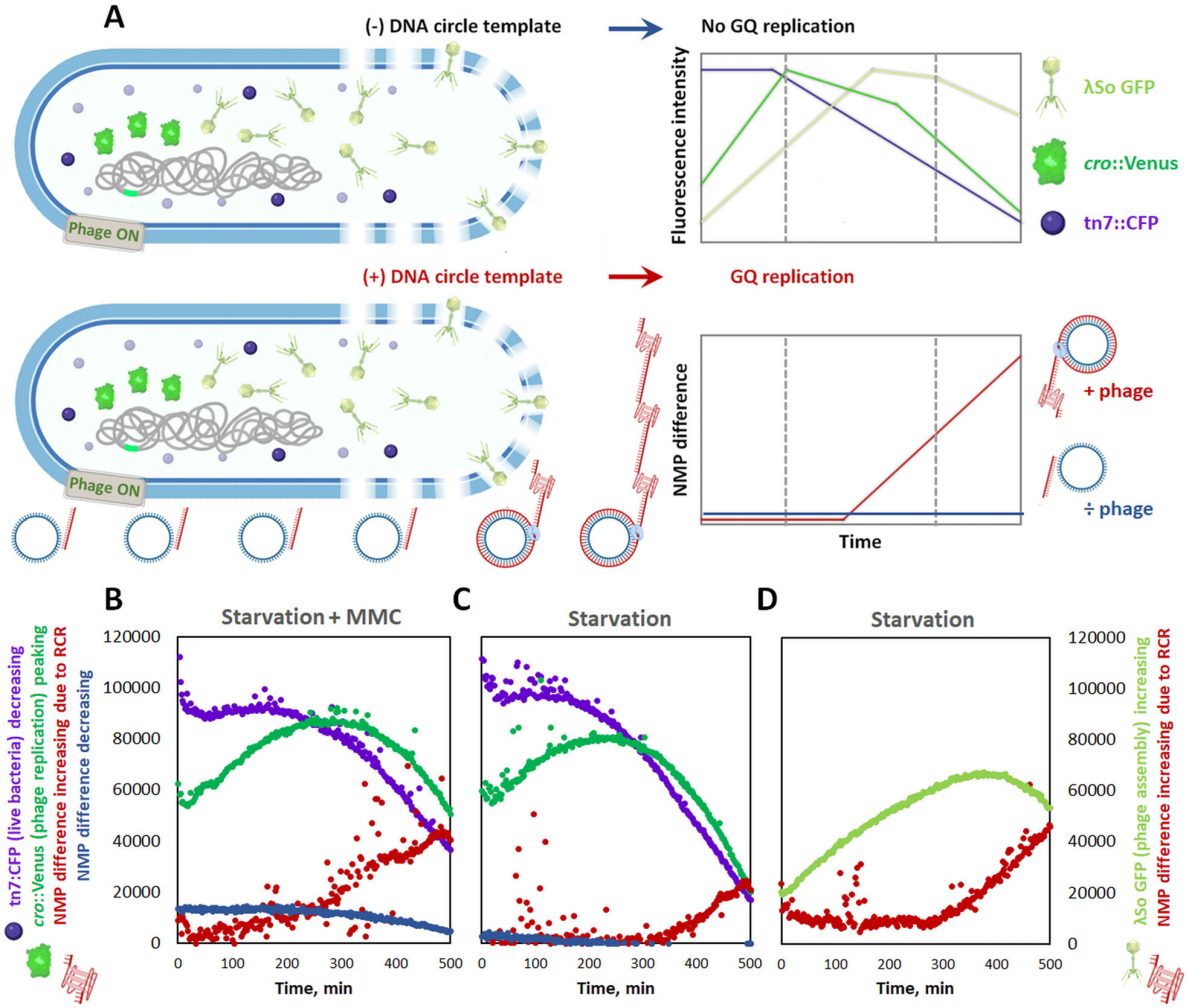
Time‐resolved phage proliferation and bacterial lysis lead to RCR and synthesis of multimeric G‐quadruplex (GQ) DNA from an exogenous template. (A) Conceptual presentation of a *S. oneidensis* cell in time. In the beginning, bacteria are intact. Upon phage proliferation and assembly, bacteria lyse and lead to extracellular replication of GQ‐DNA in the vicinity of the lysed cell. Schematic representation of phage replication detected by chromosomal *cro*::Venus fusion (the protein as well as its promoter are shown in green) in the wild‐type S2391 (live bacteria expressing *tn7*::eCFP shown in violet) and phage assembly (yellow‐green) detected by sfGFP fused to λ‐capsid in the S5558 (ΔMuSoI ΔMuSoII). Replication of the GQ‐DNA in the presence of the circular DNA T1 is detected by NMP after deducting the NMP signal of the control non‐templated cultures. The NMP difference obtained in the wild‐type and mutant (ΔλSo ΔMuSoI ΔMuSoII) cultures is shown in red and blue, respectively. (B‐D) Real‐time monitoring GQ‐DNA via NMP difference and phage proliferation via the fluorescent proteins expressed by the reporter strains. To activate phage replication in the wild‐type S2391 (*cro*::Venus fusion) reporter strain, we used mitomycin C in (B) and starvation in (C). To activate replication of phage λSo and assembly of λSo‐GFP capsids in the S5558 reporter strain, we used starvation in (D). Schematic illustration created in BioRender: Meyer, R. (2026) https://BioRender.com/7v1oxcs.

We noted that phages were active in *S. oneidensis* (wild type, *cro*::Venus) under nutrient‐limited conditions regardless of mitomycin C (MMC) (Figure 1B) or hydrogen peroxide (H_2_O_2_) (Figure S2, green). However, H_2_O_2_ is known to produce reactive oxygen species (ROS) reacting with DNA [37] and inducing chemical modifications in the DNA template and the product, and we therefore hypothesized that addition of H_2_O_2_ would inhibit extracellular RCR activity. Indeed, it resulted in a decline in the NMP fluorescence (Figure S2, red).

There is a small chance that the bacteria took up the circular ssDNA template and that RCR measured in Figure 1B–D occurred intracellularly. To provide further support for the observation of extracellular RCR activity (Figure 2A), we repeated the experiment with the exogeneous phage phi29 polymerase. Bacteria cannot take up a large protein from the extracellular environment, and we therefore hypothesized that addition of the phi29 polymerase would speed up RCR activity in the *S. oneidensis* culture, if RCR occurred extracellularly. Indeed, addition of phi29 resulted in an earlier increase in NMP fluorescence (at *t* > 100 min, Figure 2B).

**FIGURE 2:**
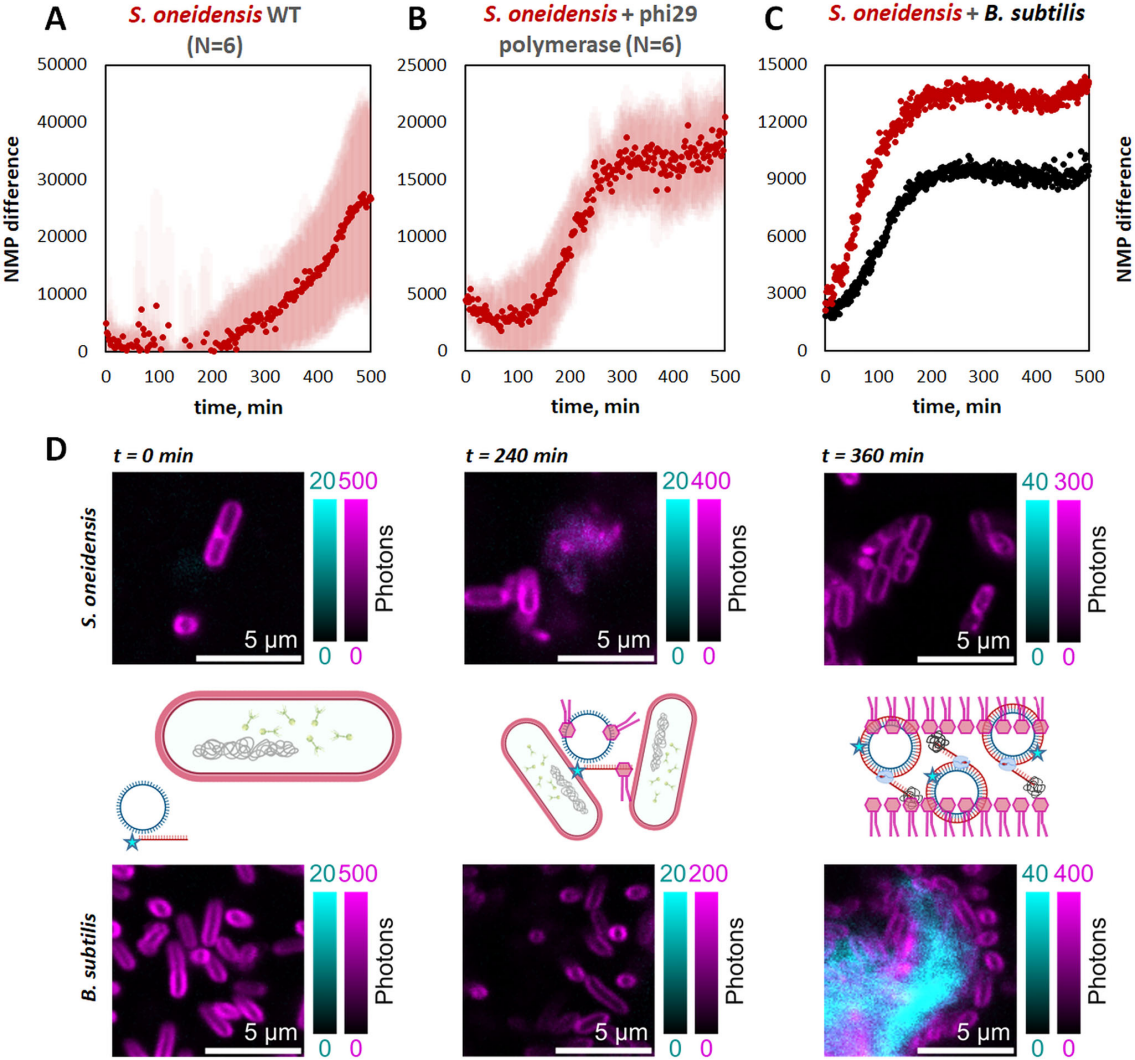
eDNA replication and assembly of superstructures is detected as increasing “NMP difference” in planktonic cultures of *S. oneidensis* and *B. subtilis*. (A) *S. oneidensis* wild‐type strains. (B) *S. oneidensis* wild‐type and ΔMuSoI ΔMuSoII strains supplemented with phi29 polymerase. Averages of six biological replicates are shown. (C) *B. subtilis* monoculture (black) and *B. subtilis* cocultured with *S. oneidensis* (red), shown as averages of two biological replicates. (D) Fluorescence imaging with single photon counting confocal microscopy of FM 4–64 lipophilic membrane dye (in magenta) and fluorescently labeled circular DNA T2 (in cyan) detected at time 0 and after 240 and 360 min of continuous incubation of bacterial cells with DNA. In the middle is the proposed scheme of DNA templates being trapped and replicated in the crowded extracellular environment. See Figure S3 for the zoomed‐out image of the superstructures formed after 360 min in *B. subtilis*. Schematic illustration created in BioRender: Meyer, R. (2026) https://BioRender.com/bdjexuv.

The phage phi29 encodes the phi29 polymerase, which is very robust in processivity [29] and which we used as a booster of extracellular RCR (Figure 2B). We wondered if the naturally present prophages of *Bacillus subtilis* can lead to an accelerated RCR. To corroborate our results from *S. oneidensis*, we performed a similar experiment in *B. subtilis*, another environmental species that harbors several prophages that use RCR: SPP1 [38], SPβ [39], phi3T [40], phi105 [41], and phi29 [42]. Figure 2C shows an immediate increase in GQ‐DNA synthesis (quantified by the “NMP difference”) in *B. subtilis* monoculture (black curve) and in *B. subtilis + S. oneidensis* coculture (red curve). These data suggest that phage replication machinery of *B. subtilis* can also facilitate extracellular GQ‐DNA synthesis from the circular template amended to the culture, and together these results support our hypothesis that RCR can occur in the extracellular space following the onset of starvation.

Ultimately, to validate the observation of extracellular RCR activity in both bacterial strains, we employed a fluorescently labeled primer (Table 1) and the T2 template with the low GC content, encoding a scrambled sequence single‐stranded R‐DNA. We detected FM 4–64 signals from the membranes of *S. oneidensis* and *B. subtilis* (magenta) and extracellular signals from the fluorescent DNA (cyan) associated with membranes of *S. oneidensis* at *t*  = 240 min and *B. subtilis* at *t* = 360 min, suggesting interaction of bacterial membranes with the circular DNA (precursor of RCR). Moreover, the superstructures detected in *B. subtilis* extracellular space at *t *> 360 min resembled elongated “sheets” or “wires.” We could not detect an uptake of the fluorescent DNA by either *S. oneidensis* or *B. subtilis* at *t *< 360 min (Figure 2D). Collectively, our NMP‐based fluorescence detection of GQ‐DNA synthesis suggests that both *S. oneidensis* and *B. subtilis* can replicate an exogenous circular DNA template after prophage activation under starvation, and this replication may take place in the extracellular environment.

### 2.2 Phage‐Mediated eDNA Synthesis in *S. oneidensis* Biofilm Results in Spherical DNA Superstructures

Our real‐time measurements of NMP fluorescence indicated production of GQ‐DNA from the exogeneous T1 template, via RCR, in planktonic bacterial cultures (Figures 1, 2). We hypothesized that concatemers of GQ‐DNA could form superstructures with insoluble salts such as PPs, often described as flowers [28], and other biopolymers in *S. oneidensis* biofilms that experience a higher degree of molecular crowding than planktonic cultures.

We inoculated *S. oneidensis* biofilms from the starved planktonic cultures into TSB media, where the surviving bacteria were cultivated in the presence of the circular template for additional 2 days to form a biofilm with GQ‐DNA products of RCR. The produced eDNA was visualized by confocal laser scanning microscopy (CLSM) using fluorescence immunolabeling with the GQ‐specific antibody BG4 [7], B‐DNA antibody AB1 (clone 35I9), the live/dead staining by DNA‐binding dyes SYTO60/TOTO‐1 and GQ‐staining by NMP [36]. Signals from BG4 and NMP staining revealed that GQ‐DNA was located inside extracellular spherical flower‐like superstructures (Figure 3A,C). Intriguingly, the DNA‐binding dyes SYTO60 and TOTO‐1 promoted Förster Resonance Energy Transfer (FRET) within these spherical superstructures (yet not inside either live or dead cells that were stained by SYTO60 and TOTO‐1, respectively, Figure 3B,E). This phenomenon was linked to simultaneous binding of both dyes and excitation of TOTO‐1 (donor), which energy was then transferred to SYTO60 (acceptor) leading to detection of SYTO60 emission. We previously showed that this FRET signal is GQ‐enriched [35]. The scrambled GC‐rich DNA (Figure S4) and double‐stranded B‐DNA (Figures S4, S5) predominantly show fluorescence from the donor, TOTO‐1. Moreover, TOTO‐1 is cell impermeable and, therefore, FRET signal is specific for eDNA unlike cell‐permeable NMP. Ultimately, we claim detection of GQ motifs in the spherical structures of up to 10 µm in size and what appeared as extracellular aggregates of these spheres using three independent GQ‐specific staining assays (Figure 3A–F). These spheres were not detected in the *S. oneidensis* WT biofilms lacking a template (Figure S6) or templated *S. oneidensis* MUT (ΔλSo ΔMuSoI ΔMuSoII) biofilms lacking prophages (Figure S7), confirming that phage replication and the exogeneous ssDNA circular template are responsible for the synthesis of GQ‐rich DNA and the formation of the superstructures. The FRET method and additional probes confirmed the extracellular nature of the synthesized DNA (Figure S5). The structures were identified to be organic, containing high weight of presumably lipids and polysaccharides (Figure 3G).

**FIGURE 3:**
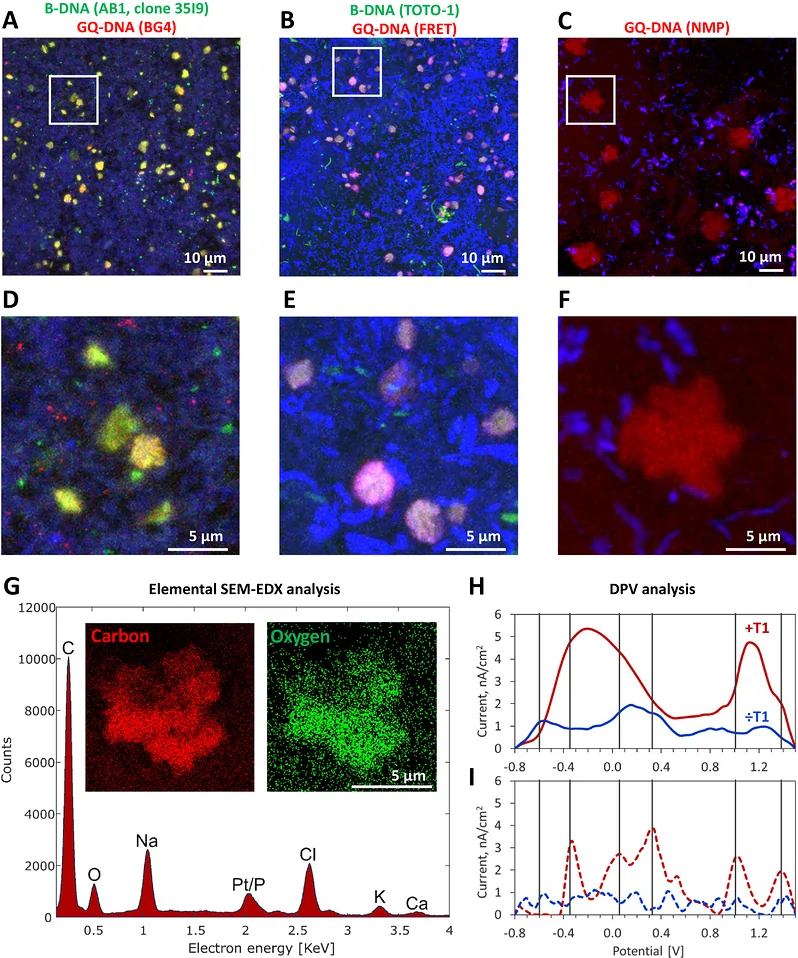
Replication of the circular DNA T1 in *S. oneidensis* biofilms leads to formation of GQ‐rich crystal‐like spherical superstructures. 3D CLSM images of the stained biofilms. (A, D) BG4 antibody (GQ, red), AB1 antibody (B‐DNA, green), and FM 4–64 (cells, blue). (B, E) DNA‐binding dyes TOTO‐1 (B‐DNA, green)/ SYTO60 (cells, blue) resulting in FRET (GQ, red). (C, F) GQ‐specific NMP dye (red) and SYTO41 (cells, blue). See Figure S5 for further details of the FRET method. (G) SEM‐EDX elemental analysis of the GQ‐DNA superstructure. (H‐I) DPV analysis of redox activity of hemin and other redox‐mediators in the biofilms of wild‐type *S. oneidensis* containing 150 nM T1 template (red curve) and non‐templated (blue curve) biofilms. DPV analyses were performed on (H) 3‐day aerobic biofilms and (I) 6‐day anaerobic biofilms.

These DNA superstructures could impact redox reactions and EET in the biofilm and potentially augment the activity of *S. oneidensis* biofilms and, for example, microbial fuel cells. We therefore detected the redox activity of DNA templated (with 150 nM T1 template) and non‐templated *S. oneidensis* biofilms grown aerobically on screen‐printed graphite electrodes. Differential pulse voltammetry (DPV) measurements detected multiple distinct peaks spanning negative to positive potential from ‐0.6 V to 1.3 V, with maximum peak currents densities of ≈ 1.8 nA/cm^2^ in a non‐templated biofilm (Figure 3H, blue curve), and a threefold increase in the current density (≈ 5.3 nA/cm^2^) in the templated biofilm (Figure 3H, red curve). In the templated biofilms, two distinct peaks spanning from ‐0.5 V to 0.4 V and from 0.9 V to 1.4 V had midpoints of ‐0.3 V and 1.15 V, respectively. We believe that these peaks correspond to convoluted redox reactions based on their broad redox potential, and that the GQ‐rich eDNA superstructures impacted these redox processes, for example, by scaffolding redox mediators (typically around ‐0.3 V) and by interaction with ROS (typically around 1 V). In the aerobic form of growth, bacteria are not forced to use EET, and the current levels are therefore low as if bacterial cells were not “connected” to the electrode. However, the DPV measurements suggest that the spherical superstructures can be the “glue” that can conduct electrons, leading to convoluted redox processes.

To omit influence of oxygen onto the redox processes, we tested these biofilms grown for additional 3‐days under anaerobic conditions (Figure 3I). Again, the templated *S. oneidensis* biofilms contained several peaks in the DPV diagram which were not present in samples of non‐templated *S. oneidensis* biofilms, indicating that DNA superstructures are redox‐active (Figure 3I). However, anoxic conditions most likely impacted on the prophage activation mechanism [43] that required ROS. After biofilms were grown anaerobically for 3‐days, the current density decreased presumably due to the lack of the ROS‐triggered prophage activation and the DPV peaks became diverged.

Although we did not investigate specific molecules eliciting the measured redox properties, we hypothesize that the overall changes in the redox activity could be due to the presence of the DNA superstructures in the biofilms with template‐directed DNA synthesis that can trap redox mediators.

### 2.3 Phage‐Mediated Intracellular DNA Synthesis in Heat‐Shocked *S. oneidensis* Biofilm

Our results strongly indicate that templated RCR occurred extracellularly, but we cannot exclude that some bacterial cells could take up the template and initiate synthesis of the GQ‐motif concatemer inside the cell. To address if intracellular synthesis from the circular template is possible, and to visualize what it looks like, we prompted uptake of the template by adding it to the bacterial suspension and exposing the bacteria to a “heat‐shock” treatment, which is commonly used to induce uptake of DNA in Gram‐negative bacteria in the presence of Ca^2+^ due to altering lipid structure [44]. Figure 4 shows biofilm formed by the “heat‐shocked” *S. oneidensis* cells stained by the intracellular SYTO60 and extracellular TOTO‐1 dyes, resulting in the GQ‐specific FRET effect. We detected SYTO60 (blue) and FRET (red) signals inside bacteria (Figure 4A) that looked different from the spherical superstructures in Figure 3A–F as they had a lipid barrier. The FRET signal was clearly confined within bacteria, and the bacterial cells became enlarged deformed and permeable for TOTO‐1 dye, possibly due to intracellular synthesis of GQ‐DNA (Figure 4B). The larger superstructures (resembling “X‐shapes”) predominantly exhibited TOTO‐1 signal (green, Figure 4A) that is known to light up (i.e., to become detectable) when bound to the canonical B‐DNA. This could be, for example, phage λSo double‐stranded DNA. SEM detected the presence of crystal‐like precipitated biopolymers inside and outside bacterial cells and the elemental analysis of the precipitated DNA resulting from the “intracellular” replication (Figure 4C–E) was similar to the spherical superstructures resulting from the “extracellular” replication of GQ‐DNA (Figure 3G). From these experiments, we concluded that “heat‐shocked” *S. oneidensis* in the presence of the T1 template produced an enlarged and deformed phenotype of cells, indicating uptake of the circular template and the intracellular mode of GQ replication.

**FIGURE 4:**
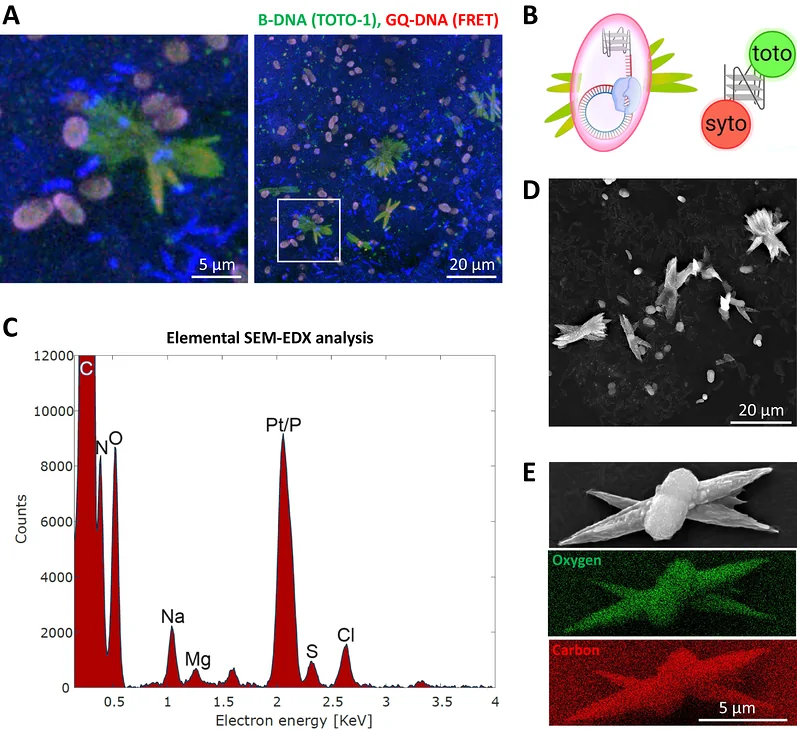
Intracellular DNA synthesis and extracellular shapes formed in DNA‐templated “heat‐shocked” monospecies *S. oneidensis* MR‐1 in TSB‐KCl, imaged by (A, B) CLSM and (C–E) SEM‐EDX. The cartoon shows bacterial cells from (A) stained by the DNA‐binding dyes SYTO60 (acceptor excitation, acceptor emission in blue) and TOTO‐1 (donor excitation, donor emission in green) resulting in the FRET (donor excitation, acceptor emission in red). The difference from Figure 3 is the phenotype of the resulting templated biofilms, in which FRET‐stained DNA products of RCR appear to be inside enlarged and deformed bacterial cells. See Figure S5 for further details of the FRET method. Schematic illustration created in BioRender: Meyer, R. (2026) https://BioRender.com/4s9xc6n, https://BioRender.com/d8ulv65.

### 2.4 Phage‐Mediated eDNA Synthesis in *B. subtilis* Biofilm Results in Wire‐Like Superstructures

We have now provided proof‐of‐concept for eDNA synthesis and crystallization of redox‐active DNA superstructures in *S. oneidensis* biofilms. In principle, this could be a generic concept that can be applied in other microorganisms that encode prophages with polymerases capable of RCR. To demonstrate the concept in another species, we showed that *B. subtilis* encoding prophages SPP/SPβ followed a similar but faster kinetics of RCR detected by the “NMP difference” (Figure 2C), indicating synthesis of GQ‐DNA. Next, we wondered if *B. subtilis* also produced GQ‐DNA superstructures in biofilms. Using starved *B. subtilis* to inoculate biofilms, we observed elongated wire‐like superstructures in templated biofilms. In 3‐day biofilms, wires occurred in both templated and non‐templated biofilms, which could be the result of phage activity and replication of phage and spurious DNA. Therefore, these wire‐like superstructures could contain phage or genomic DNA in addition to the programmed GQ‐sequence. To ultimately differentiate the products of the templated RCR from other DNA replication, we detected peroxidase activity of the GQ‐DNAzymes [45] synthesized from T1 using fluorescent tyramide signal amplification (TSA) (Figures 5A and S8A in green). In this assay, hemin bound to GQ motifs catalyzes reduction of hydrogen peroxide and covalent addition of the tyramide residues to macromolecules in the vicinity of the catalyst. Furthermore, we detected binding of a fluorescently labeled oligonucleotide cDNA (Figures 5B and S8B in blue) that is complementary to the DNA chain synthesized via RCR from either T1 or T2 template (Table 1). Figure 5 shows a DNA “wire” produced in *B. subtilis* biofilm containing both the tyramide and cDNA signals and visualized using CLSM and SEM.

**FIGURE 5:**
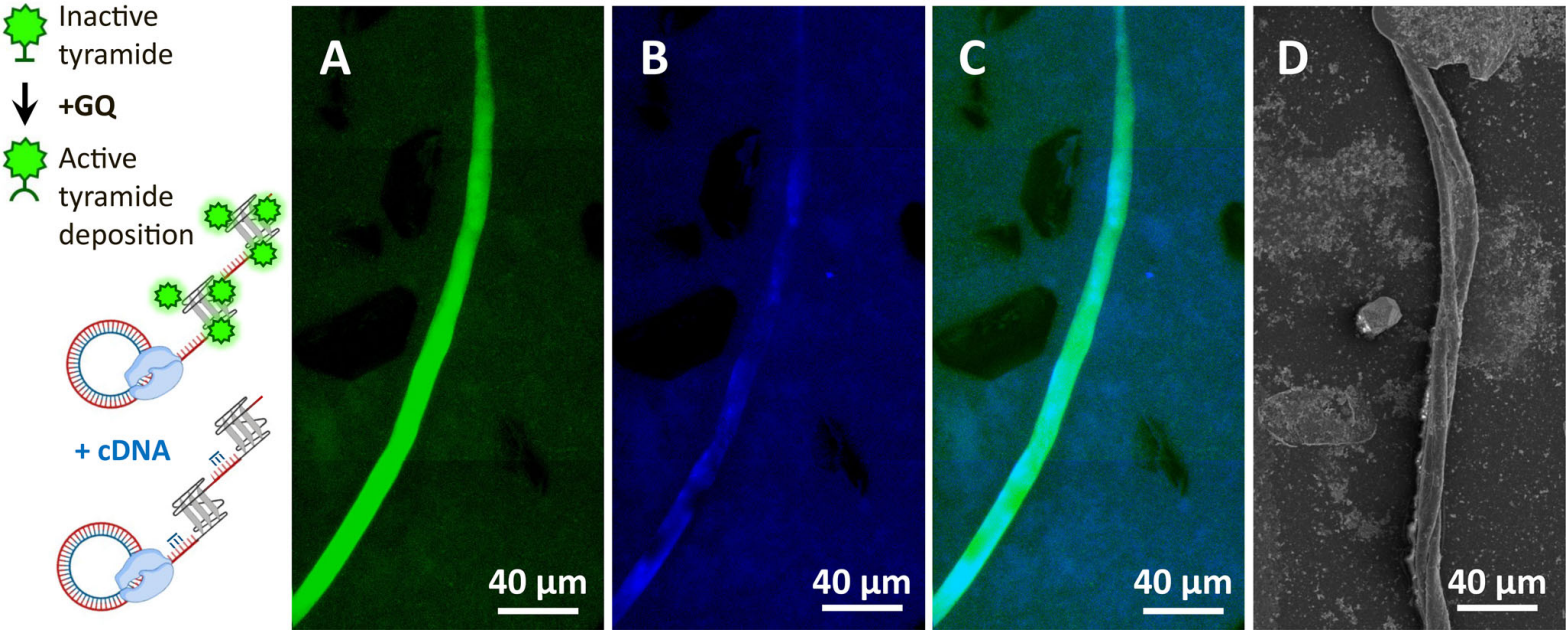
Replication of the circular DNA T1 in *B. subtilis* biofilms leads to formation of crystal‐like “wires”. Schematics show DNAzyme‐mediated deposition of fluorescent tyramide (green) in the vicinity of the GQ‐DNAzymes followed by hybridization and retention of fluorescent cDNA (blue). GQ‐DNA superstructures were synthesized in 3‐day *B. subtilis* biofilms in TSB‐NaCl with exogenous T1 (150 nM), simultaneously labeled by (A) tyramide residues and (B) cDNA and visualized by CLSM. (C) Merged CLSM image and (D) SEM image of a twisting DNA wire. See Figure S8 for additional CLSM images as well as SEM‐EDX elemental analysis. Schematic illustration created in BioRender: Meyer, R. (2026) https://BioRender.com/68plb9t.

Two distinct strains triggered replication of DNA from a template provided exogenously, resulting in wire‐like superstructures in *B. subtilis* and in spherical flower‐like superstructures in *S. oneidensis*. We wondered if these phenotypes remain in the consortium containing dual species. Using the FRET signal from TOTO‐1 / SYTO60 staining (Figure S9) and NMP staining (Figure S10) to detect GQ‐DNA, we confirmed the presence of GQ‐DNA in wires formed after day 3 of dual species biofilm growth in *S. oneidensis* (*tn7*::CFP, in blue) cocultured with *B. subtilis*. We, furthermore, noticed that the “wires” had CFP signal, suggesting that the CFP from *S. oneidensis* aggregated and integrated into the “wires.” Thus, the superstructures comprised of GQ‐DNA self‐assembled with other biopolymers originating from both microorganisms. Next, we applied TSA and cDNA labeling to ultimately validate the function and the sequence of the RCR products within those “wires.”

As we cannot exclude natural peroxidase activity present in such giant supramolecular systems, we also recruited an alternative template T2, resulting in a RCR product that is complementary to the cDNA probe but does not form GQ (Table 1). Figure 6A–C show examples of the DNA “wires” detected in the 3‐day biofilms of *B. subtilis* + *S. oneidensis* with the original T1‐template (A), an alternative T2‐template (B) or without template (C). As we hypothesized, the tyramide signal was detected only in the wires, in which GQ‐DNA was synthesized from T1 template (due to either the DNAzyme activity of GQ/ hemin or GQ scaffolding other redox molecules). No tyramide signal was found in the wires enriched with R‐DNA synthesized from T2 template.

**FIGURE 6:**
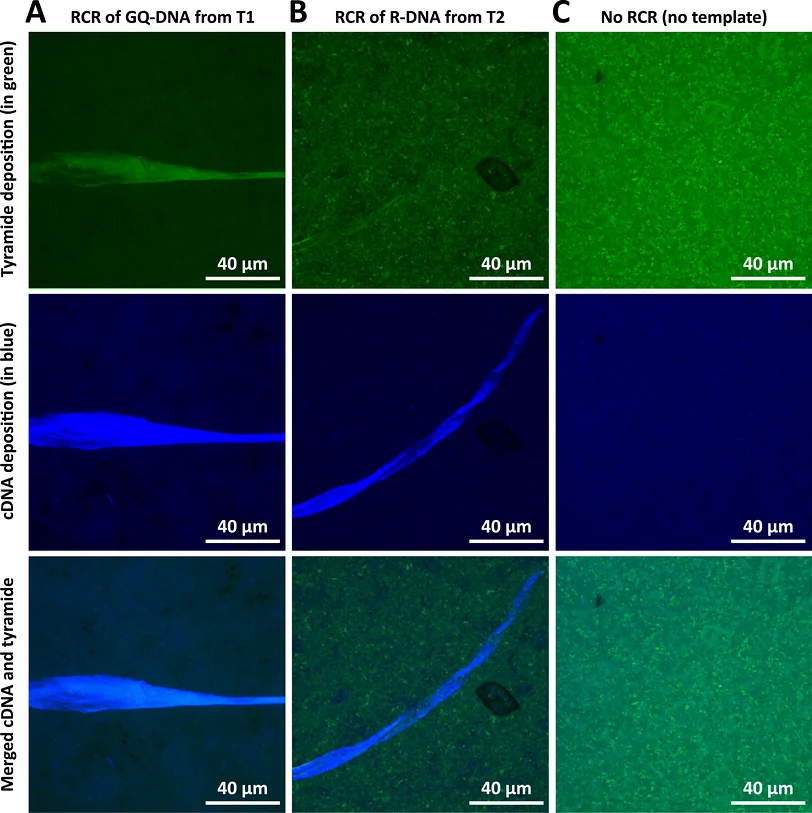
The synthesis of DNA from circular DNA templates in cocultured *B. subtilis + S. oneidensis* leads to formation of crystal‐like “wires” with distinct catalytic function. CLSM images of the wire‐like superstructures labeled using elaborated tyramide signal amplification (TSA) and cDNA labeling. Biofilms were doped with (A) 150 nM circular DNA template T1 for GQ‐DNA and (B) 150 nM circular DNA template T2 for R‐DNA production. (C) Biofilms were grown without addition of any template. The 3‐day biofilms were stained by tyramide residues (Alexa488) in the presence of 0.5 mM ATP and 0.2% H_2_O_2_ following addition of 5 µM cDNA (5’‐Alexa647). See Figure 5 for the assay illustrations. (A), (B), and (C) refer to three locations in three distinct samples. For further CLSM imaging of GQ‐DNA wires in the T1‐templated *S. oneidensis* + *B. subtilis* biofilms see Figure S9 (stained by SYTO60/ TOTO‐1 FRET pair) and Figure S10 (stained by NMP).

We propose that the wire‐like superstructures are formed from a DNA concatemer produced by RCR from each of the circular DNA templates, which were added during biofilm formation, and that the replication requires activation of prophages in both *S. oneidensis* and *B. subtilis*. No spherical structures were detected in the dual‐species biofilms, suggesting a leading role of *B. subtilis* in multispecies biofilm, which also agrees with the faster rate of lytic RCR detected in Figure 2. Thus, the replication machinery of bacteriophages in *B. subtilis* may be superior to replication of phage λSo in *S. oneidensis*. The carbon‐rich composition of the “wires” (Figure S8) implies that such superstructures are formed in response to cellular lysis, like we propose in Figure 2.

## 3 Discussion and Concluding Remarks

eDNA plays a predominant role in proliferation of various microorganisms including genus *Shewanella* and *Bacillus* [46]. *S. oneidensis* is being harnessed in various biotechnological applications ranging from environmental bioremediation, metal recovery, and material synthesis to bioenergy generation [47]. *B. subtilis* is being harnessed for several applications, such as microbial fuel cells [48] and even a battery [49]. However, both species produce biofilms that are poor in eDNA, where eDNA is predominantly existing in the relics of dead cells. Efforts to encapsulate *S. oneidensis* into layers of DNA give eDNA a new role to facilitate electric current generated by cells, suggesting eDNA may be a key to construct microbial fuel cells [11].

While previous studies of eDNA production in biofilms focus on how eDNA is released from bacterial cells [1], we now show that eDNA can be synthesized from nucleotides directly in the extracellular matrix by phage polymerases. This has implications for how eDNA might be organized in the extracellular matrix, as formation of DNA via this mechanism can impact eDNA crosslinking, eDNA conformation, and eDNA‐protein interactions in the biofilm. Hence, our findings reveal an overlooked dimension of phage‐bacteria interactions where prophages orchestrate extracellular eDNA synthesis. Role of starvation in prophage activation is intriguing: some prophages were reported to activate spontaneously upon starvation‐induced sporulation [40, 50], whereas prophage λ was reported to remain in its lysogenic state under cell starvation [51, 52]. We present a new universal method for detecting prophage activity in two distinct bacterial strains by monitoring rolling circle replication of an exogeneous template. Using this method, we show that starvation‐induced prophage replication was detected in *S. oneidensis* and *B. subtilis* planktonic cultures. The timing of prophage‐induced RCR agrees well with Thöneböhn et al. reporting a 100 min lag phase before phage assembly and cellular burst [18]. Thus, short‐term starvation is likely to induce the phage proliferation at the prolonged lag phase, and this could be a generic and sustainable way to activate prophage rolling circle replication.

Hundreds of kilobases are generated using RCR from a DNA template by a robust phage polymerase in a controlled cell‐free environment [29]. By designing the circular template, the DNA products of RCR are programmed to fold into a desired GQ conformation. GQs are highly in demand as these structures can bind a plethora of molecules including redox‐active riboflavin [5], hemin [53], cytochrome [54], yielding modular functional electrocatalytic networks [55] and potential for hierarchical assembly. Multiple GQ‐motifs were shown to assemble intermolecularly resulting in 10–400 nm wire‐like nanostructures of ≈2 nm in height and ≈20 nm in width [56], making them ideal as building blocks to assemble complex superstructures and further organize various bio‐ and inorganic molecules. GQ wires containing a large number of stacked guanine tetrads can provide a more efficient π‐stacking compared to B‐DNA [57], yielding a scaffold and ionic pathways for biobatteries [58]. Furthermore, GQ‐DNAzymes were demonstrated to break graphene oxide [10], suggesting there is an overlooked power of DNA to be harvested. Further studies need to address constructing GQ‐enriched functional superstructures from various DNA templates (with different number of CCC stretches and different distances between them) yielding DNA concatemers with tailored folding and spacings between GQs or other, for example, pH‐responsive motifs [59]. Indeed, the advantage of using RCR is that circular DNA templates can be easily tweaked to control, for example, EET or catalytic activity of GQ‐DNA synthesized in situ.

We prompted the synthesis of GQ‐DNA concatemers that folded into superstructures with remarkable morphological diversity that appears to be species‐dependent. The resulting shapes, which are spherical for *S. oneidensis* and wire‐like for *B. subtilis*, can be explained in terms of self‐assembly of DNA with bacterial phospholipids (cartoon in Figure 2D) and other macromolecules leading to formation of various amphiphilic complexes [60]. Similar wire‐like structures were detected in staphylococcal biofilms, a culprit of implant‐associated infections (*S. epidermidis* 1457, Figure S11). We therefore encourage microbiologists and clinicians to collect such data due to increasing application of phage treatment in biomedicine.

We demonstrated detection of the GQ superstructures within the “flowers” and “wires” in all T1 templated biofilms of *S. oneidensis* and *B. subtilis*, respectively, using the FRET pair of dyes TOTO‐1 and SYTO60, the GQ‐specific dye NMP, the complementary cDNA probe and ultimately the peroxidase‐like TSA (Table S1). Furthermore, we used T2 to produce R‐DNA, a non‐GQ control, that was stained positively using the cDNA probe complementary to the conservative region in the GQ‐ and R‐DNA concatemers. However, only GQ‐rich T1‐derived DNA “wires” had the catalytic peroxidase‐like activity. We therefore propose that our approach to DNA synthesis is generic and is programmed by the circular DNA template that we designed for replication of GQ‐DNA and random (non‐GQ) R‐DNA (Table 1). Nevertheless, we acknowledge that our observations are limited to the synthesis directed by T1 and T2 templates and to the YES/NO readout of the biosynthetic DNA in the extracellular superstructures. Future studies of the EET and catalytic activity of these DNA complexes and superstructures should test series of products synthesized from different templates. The qualities of the DNA superstructures can be tweaked via sequence design of the template directing the synthesis of DNA via RCR.

Electromicrobiological processes are often limited by the electron transfer processes between the electrode and the associated microorganisms. New approaches to harness eDNA for building and enriching biofilms are being investigated. For example, GQ‐rich eRNA is shown to drive electromethanogenesis in a methanogenic archaeon [9]. Therefore, enriching environmental biofilms with GQ‐DNA can be a way to scaffold bacterial redox‐active molecules, maintaining them in spatial arrangement for a robust EET [6, 55]. Moreover, due to the formation of DNA superstructures in *S. oneidensis* biofilms, we detected an increased level in current in the DPV analysis suggesting enhanced and convoluted redox‐active processes, which will be beneficial for the applications in bioelectronics [61, 62], biocatalysis [10, 63] and environmental biotech [64]. The magnitude of current densities observed in this study could have been reduced by the culture method applied here.

To our knowledge, this is the first time rolling circle replication has been orchestrated in a complex multicellular environment, where synthesis of DNA may be slow but unlimited in resources. Further analysis is required to understand organization of various biopolymers within the superstructures, as their formation is most likely dominated by lipids, proteins, polysaccharides and other macromolecules derived from bacterial cells. This may result in inhibition of RCR and the formation of the non‐templated spherical and wire‐like composites. The interplay between various macromolecules within the superstructures may impact the electrocatalytic properties of GQ‐DNA by either mis‐folding these structures or shielding them from the electron acceptors. These concerns can be addressed by the addition of special enzymes that can selectively disentangle biopolymers in the superstructures.

## 4 Materials and Methods

### 4.1 DNA Oligonucleotides

DNA oligonucleotide sequences (Table 1) were purchased from Integrated DNA Technologies and stored as 100 µM aliquots (“LabReady” in 1 mM Tris‐EDTA buffer) at −20°C. The circularizing DNA templates (ultramers) were 5’‐phosphorylated. The fluorescently labeled oligonucleotides were purified via PAGE.

### 4.2 Dyes

TOTO‐1, SYTO41, and SYTO60 in DMSO (Thermo Fisher Scientific) were aliquoted in Milli‐Q water at 250 µM concentration and stored at ‐20 °C. The GQ‐specific NMP (LivChem), 20 mM in DMSO, was aliquoted at 250 µM concentration in Milli‐Q water. The membrane‐binding dye FM 4–64 (Thermo Fisher Scientific) was diluted 1 g/L in milliQ water. NMP and FM 4–64 were stored at 4°C. The dyes were further diluted in reaction buffer to their working concentration.

### 4.3 Synthetic DNA Ligation

DNA universal primer (5 µM; Table 1) and one of the linear templates T1 or T2 (4 µM; Table 1) were gently mixed in 1× Ampligase buffer (BioSearch; Cat.No. A3202K, A1905B) and Ampligase (150 u/mL; BioSearch; Cat.No. A3202K). The circularization of DNA templates T1 and T2 (Table 1) was obtained by annealing of 5’‐phosphorylated single‐stranded DNA “padlock probes” onto complementary DNA target at 60°C (20 min), 55°C (20 min), and 50°C (20 min) and simultaneous ligation using 150 u/mL Ampligase (Biosearch) [29]. Subsequently, the ligated DNA cooled down gradually to room temperature for another hour. The ligated DNA were applied for rolling circle replication of multimeric GQ and random DNA from the two respective DNA templates (Table 1).

### 4.4 Synthetic Rolling Circle Replication

Synthesis of GQ‐DNA was achieved via RCR from template T1 using phi29 polymerase [29]. Reagents: circular DNA (4 µM), phi29 polymerase (10 u/µL; Thermo Fisher; Cat.No. EP0094), phi29 buffer (10×; Thermo Fisher; Cat.No. B62), BSA (20 mg/mL; Merck), dNTPs (100 mM; NEB; Cat.No. N0446S), NMP (250 µM; LivChem). A set of 220 µM dATP, dTTP, dCTP and 880 µM dGTP was mixed with 12.5 nM, 50 nM and 100 nM circular DNA in 1X phi29 buffer (33 mM Tris‐acetate, 10 mM magnesium acetate, 66 mM potassium acetate, 0.1% Tween 20, 0.2 mg/mL BSA, 1 mM DTT, pH 7.9) and 40 µM NMP in MilliQ water. The mixture was split into 3x3 array: (1) three samples containing three DNA concentrations and phi29 polymerase, (2) three samples containing phi29 polymerase but no DNA, (3) three samples containing three DNA concentrations and no phi29 polymerase. RCR was triggered by adding phi29 polymerase to the groups (1)+(2) and all samples were placed into CLARIOstar plate reader at 37°C. The excitation/emission was 405/615 nm for NMP.

### 4.5 Bacterial Strains Used for Prophage Induction

Strain *B. subtilis* (WT) was acquired from DSMZ culture collection (DSM‐10). Strains *S. oneidensis* MR‐1 WT, S2391 (*tn7*::CFP, *cro*::Venus) [13], and MUT (ΔλSo ΔMuSoI ΔMuSoII) [65] were published previously. To construct a reporter for LambdaSo capsid production in *S. oneidensis* MR‐1 (ΔMuSoI ΔMuSoII), we integrated a second copy of the capsid‐encoding gene SO_2963 fused to *sfgfp* into the LambdaSo chromosome by sequential crossover. To avoid subsequent homologous recombination between the two SO_2963 genes and loss of one of the copies, we used a synthetic version of the gene (SO_2963EC). This version was synthesized by Genscript (Rijswijk, NL) and was codon‐optimized for *Escherichia* *coli* (see sequence below, delivered in pUC57 (Thermo Fisher Scientific), resulting in a gene with 75% DNA sequence identity to the original LambdaSo gene. To construct the hybrid gene by Gibson assembly [66], the appropriate DNA fragments were amplified by PCR using the given primers (Table 2).

**TABLE 2:** Primers for constructing hybrid gene SO_2963 fused to *sfGFP* in *S. oneidensis* S5558.

	Primers	Template
Upstream fragment	MK345: GAATTCGTGGATCCAGATcagctgttatacggcaacaaca MK346: ATTTGCCCTCCTGGCAGCttaggcaaaagtaaagttacccttaaca	*S. oneidensis* chromosomal DNA
SO_2963EC fragment	MK347: ATTTGCCCTCCTGGCAGCTTAGGCAAAAGTAAAGTTACCCTTAACA MK348: GTCTCCCATGCTGCCGCCGGCAAAGGTAAAATTCCCCTTGA	pUC57::SO2963EC
sfGFP fragment	MK349: ACCTTTGCCGGCGGCAGCATGGGAGACCGCGGTCCC MK350: GTTAACCGATTTATGCTGTTAGTGGTGGTGGTGGTGG	S. *putrefaciens* chromosomal DNA [67]
Downstream fragment	MK351: CACCACCACCACCACTAACAGCATAAATCGGTTAACTGATAACTAAAC MK352: CAAGCTTCTCTGCAGGATCGTCCTCTGCCCCATCAA	*S. oneidensis* chromosomal DNA

The PCR reactions contained HF buffer (5x; Fisher Scientific; Cat.No. F518L), dNTP mix (stock concentration 10 mM each; Roth; Cat.No. K039.1), primers (Microsynth; 50 µM each), Phusion polymerase (2U; Fisher Scientific; Cat.No. F530L) and 50 ng template DNA. Denaturation was carried out at 95°C, annealing was done at 57°C, elongation at 72°C, each for 30 s. The cycle was repeated 30 times.

The obtained fragments were gel‐purified (NucleoSpinTM Gel and PCR Clean‐up Kit; Fisher Scientific; Cat.No. 11992242) and then fused with each other and EcoRV‐digested pNTPS138‐R6K [68] (EcoRV obtained from NEB; Cat.No. R3195). The reaction contained HiFi DNA Assembly Master Mix (5x; NEB; Cat.No. E2621L), 50 ng vector DNA and about 150–300 ng PCR derived fragments. The mixture was incubated for 1 h at 50°C and then directly used for transformation into *E. coli* DH5alpha‐LambdaPir [69]. The insert of the resultant plasmid was sequenced by SeqLab for confirmation using standard M13 primers. The correct vector was then transformed into the strain *E. coli* WM3064 (W. Metcalf, Urbana, Champagne), from which the plasmid was conjugated into the recipient *S. oneidensis* (S1422; lacking the prophages MuSo [65]). Selection for primary and secondary crossover was carried out as previously described [68]. Candidate *S. oneidensis* colonies were checked by fluorescence microscopy and PCR. The resultant strain S5558 produced active LambdaSo phage particles and displayed green fluorescence upon production of the capsid.

### 4.6 Sequence of SO_2963EC

ATGCCGAACCCGAACTTTGAAAAACAAATAGAGGAGTTAGGCGTTAACTTGACCAAGATCGGTGACCAGATTAAGAGCGCCGCAGAGGAAACCAATAAGCAGATAAAAGCCTCAGGCGAAATGCATGCTGAAACCAGAGATAAGGTAGACAAGTTGCTGAGCGAACAAGGTGCGTTACAAGCACGTCTTCAAGAGGCGGAACAGAAGCTGTTAAAAGGAGCGCAGTCCAACCAGCAGGAGAGAGAGCAGTCGATCGGCGAACGGGTCGTTAACGACAAGGAGATGGAGGGAGTTTCATCATCCTTCCGTGGGAGTCGTCGTGTGGGAATGCCGCGTAGTGCGATAACAAGCGCGACAGGCAGTGGCGCGGGCCTGGTCCGGCCCGATCGGATGGCGGGAATAGTTGCTGGACCGGAACGTCGTCTTACCATTCGTGACCTGATCGCACCCGGGGAGACTGAATCGAATACCATCGAGTACGTCAAAGAGACCGGCTTTACTAACAACGCGGCGCCCGTGGCCGAAGGGGCCGCCAAACCTTATTCTGAGATCACTTTTGGGTTAGTATCCGCTGGTGTCCGCACAATAGCTCACTTGTTCAAAGCCTCCCGCCAGATTCTTGATGATAGCAAACAATTACAGAGTTTCATAAATGCCCGTGCCCGTTATGGGTTGATGCTGAAGGAGGAAGTTCAGCTTCTTTACGGTAATAATACAGGAGCGAATCTGCACGGAATTATGTTACAAGCTAGCGCCTACGTCAAACCGACAGGGGCGACTGTAGACACTGAGCAGCACATTGACCGCATCCGTCTTGCATTGTTACAGGCAGCCTTAGCTGAGTACGCAGCAGATGGAATAGTATTAAACCCAATCGACTGGGCGGTAATAGAAACTTTGAAAGACTCGAATAAGAATTATCTTATTGGTAAACCCCAAGGGCAAACCGTCCCAACTTTATGGAATCGGCCAGTAGTCGAAACACAGTCAATTGTCCAAAATGAGTTTTTGGTCGGCGCGTTTCAGATGGGTGCGCAAATATACGACCGCATGGATATAGAAGTACTGATTTCAACTGAAAATGACAAAGACTTTGAAAATAATATGGTATCAATACGTGCTGAAGAGCGCCTTGCTCTGGCTGTGTACCGTCCGGAGGCTTTCGTCAAGGGGAATTTTACCTTTGCCTAA

### 4.7 Prophage Activation in Planktonic Cultures and Biofilms

*S. oneidensis* and *B. subtilis* strains were streaked onto LB agar plates and incubated for 24 h at room temperature and 24 h at 30°C. A single colony was transferred to LB media to obtain overnight cultures at 30°C reaching stationary/starved phase (OD ≈2.5). Biofilms of *S. oneidensis* were grown in TSB media with 100 mM NaCl or KCl and optionally 20 µM iron sulphate or 20 µM mitomycin C or 0.2% H_2_O_2_ (to test prophage induction/ activation).

A buffer was developed for prophage induction in planktonic bacteria: 0.5× phi29 buffer (16.5 mM Tris‐acetate, 2 mM (CH_3_COO)_2_Mg, 33 mM CH_3_COOK, 0.05% Tween 20, 0.5 mM DTT, pH 7.9) containing optionally FeSO_4_ (20 µM) or MMC (20 µM) in milliQ. Bacterial cells were harvested from overnight culture, resuspended to OD 5 in the buffer, and incubated in Ibidi 96‐well plate for 24 h at 30°C to induce prophage‐associated rolling circle replication. The buffer was subsequently replaced with TSB media (with 100 mM NaCl or KCl), and the biofilms were grown for 48 h with and without an exogenous 20–200 nM circular DNA template.

To boost possible intracellular replication, a “heat‐shock” step was introduced, in which bacterial cells were pre‐incubated at 42°C (2 min) and 5°C (1 h) in the working buffer prior to the step of prophage activation and replication. This led to crystallization processes inside deformed cells of *S. oneidensis* and formation of larger crystal shapes, which were very distinct from spherical superstructures.

### 4.8 Fluorescence Microscopy Imaging of Biofilms

Fluorescence microscopy was conducted using two confocal microscopes—the single‐photon counting Luminosa microscope (PicoQuant) and Zeiss LSM700. Samples were measured in μ‐Slide 8 well IbiTreat slides (Ibidi; Cat.No.: 80826) and in 96‐well IbiTreat plates (Ibidi; Cat.No: 89626), respectively, at room temperature.

The Luminosa microscope is based on the inverted Olympus IX73 microscope with a 60x PlanApochromat water immersion objective (NA = 1.2) and Single Photon Avalanche Diode (SPAD) detectors. The fluorescence images were scanned in two different sizes: (1) 10x10 µm of 128x128 pixels with a pixel size of 78 nm and pixel dwell time of 20 µs for imaging the uptake of DNA template T2 in planktonic cultures and (2) 200x200 µm of 1024x1024 pixels with a pixel size of 195 nm and pixel dwell time of 5 µs to obtain fluorescence‐based imaging of biofilms.

The Zeiss LSM700 microscope is equipped with a 40×/NA1.4 and 63×/NA1.4 Plan‐Apochromat objective using 405, 488 and 639 nm excitation (MBS 405/488/555/639). The Zeiss LSM700 images were scanned as 2D as well as 3D in sizes of (1) 160x160 µm and (2) 100x100 µm of 1024x1024 pixels.

### 4.9 Real‐Time Detection of Phage Proliferation and Rolling Circle Replication in Planktonic Cultures

Bacterial cells were harvested from overnight cultures, resuspended to OD 5 in the working buffer (16.5 mM Tris‐acetate, 2 mM (CH_3_COO)_2_Mg, 33 mM CH_3_COOK, 0.05% Tween 20, 0.5 mM DTT, pH 7.9) with and without circular T1‐template.

We used *B. subtilis* and various *S. oneidensis* reporter strains and 40 µM NMP to measure molecular processes of phage DNA replication and capsid assembly and extracellular synthesis of GQ‐DNA, respectively. Initially, we obtained overnight cultures of these strains in LB media (16‐20 h, 30°C, aerobic, 180 rpm). Next, we upconcentrated the cells to OD 5 in the working buffer. The harvested cells were transformed into 96‐well plate and incubated for 8 h at 30°C in a CLARIOstar plate reader using following excitation/emission settings: 405/615 nm (NMP), 435/485 nm (*S. oneidensis* CFP), 480/520 nm (*S. oneidensis* GFP and Venus protein). To quantify the NMP signal associated with the GQ‐DNA synthesis from GQ‐DNA template, we used “NMP difference” obtained by subtracting the NMP signal in “planktonic cells ÷ DNA” from the NMP signal in “planktonic cells + DNA”. To quantify the NMP signal associated with the GQ‐DNA synthesis by exogeneous phi29 polymerase, the “NMP difference” was obtained by subtracting the NMP signal in “planktonic cells + DNA ÷ phi29” (control without exogeneous polymerase) from the NMP signal in “planktonic cells + DNA + phi29”. The fluorescence of CFP, Venus, and GFP and the “NMP difference” were plotted versus time (2 min data pitch).

The standard deviations were calculated using Microsoft Excel based on 6 biological replicates in Figure 2A and B. In Figure 2C, averages of two biological replicates are shown.

We used the single photon counting Luminosa microscope to detect an uptake of the T2 template circularized onto the fluorescently labeled universal Alexa405‐primer (Table 1) in planktonic cultures of the wild‐type *S. oneidensis* and *B. subtilis* strains. DNA location was monitored after 0, 240, and 360 min of continuous incubation of *S. oneidensis* and, separately, *B. subtilis* bacterial cells with DNA. For detection of the lipophilic membrane dye FM 4–64 (Thermo Fisher Scientific) and Alexa405‐DNA, the dyes were excited using the 488 nm laser (LDH‐D‐C‐485S, around 0.1‐0.5 µW) and 405 nm laser (LDH‐D‐C‐405S, around 0.1‐0.5 µW), respectively. Alexa405 was detected around 430–460 nm (BP445/30) whereas FM 4–64 was detected around 663–735 nm (BP700/75). A laser repetition rate of 40 MHz was used. FM 4–64 stained cellular membranes and exogeneous Alexa405‐DNA were visualized in magenta and cyan, respectively. The magenta channel was kept at a maximum of 200–500 photon counts, and the cyan channel was set to be between 20 and 40 photon counts, specified with an intensity scale for each image shown.

### 4.10 End‐Point Detection of Rolling Circle Replication Products in Biofilms Using DNA‐Binding Antibodies

Biofilms were immunolabeled in 3% BSA. After a 5 min blocking step with 3% BSA in 1 × PBS, in vitro biofilms in separate microwells were incubated with 60 µL solutions of Atto488‐BG4 antibody (10 mg/L; Absolute antibodies) in the 3% BSA for 75 min. Second, we added a 60 µL solution of mouse AB1 antibody (clone 35I9; 5 mg/L; Abcam) in the 3% BSA and continued incubation of the biofilms with the two antibodies for another 75 min. Third, after washing the biofilms with 120 µL of the 3% BSA, the biofilms were incubated with 60 µL solution of anti‐mouse CF405S‐AB2 antibody (10 mg/L; Merck) in the 3% BSA. The biofilms were washed in the 3% BSA and, finally, stained in a 10 mg/L solution of FM4‐64 in 100 mM NaCl. The stained biofilms were visualized by CLSM using a 63× oil‐immersed objective (Zeiss LSM700) showing BG4 in red, AB1‐AB2 in green, and FM 4–64 in blue.

### 4.11 End‐Point Detection of Rolling Circle Replication Products in Biofilms Using DNA‐Binding Dyes and FRET

Combinations of DNA‐binding dyes NMP + SYTO41 (for a quick staining) or SYTO60 + TOTO‐1 (for FRET staining) were applied to biofilms in 100 mM NaCl solutions just before imaging.

In vitro biofilms of *B. subtilis* + *S. oneidensis* S2391 (live cells = CFP, ex. 405 nm, em. 300–550 nm, gain 1000) were incubated with 60 µL solution of 25 µM NMP (ex. 405 nm, em. 600–800 nm, gain 1000) to visualize GQ‐DNA. In vitro biofilms of *S. oneidensis* (wild‐type MR‐1) were incubated with 60 µL solution of 25 µM NMP (ex. 405 nm, em. 600–800 nm, gain 1000) and 12.5 µM SYTO41 (ex. 405 nm, em. 400–600 nm, gain 650) to visualize GQ‐DNA and intracellular DNA in the live cells, respectively. GQ‐DNA and live cells were presented in red and blue, respectively. In vitro biofilms of *S. oneidensis* and *B. subtilis* (both single and multispecies) were incubated with 60 µL solution of 2.5 µM TOTO‐1 (ex. 488 nm, em. 400–630 nm, gain 900) and 12.5 µM SYTO60 (ex. 639 nm, em. 630–800 nm, gain 900) to visualize total eDNA and live cells, respectively. The eDNA and live cells were presented in green and blue, respectively. Since the two dyes bind GQ‐DNA [35], we used FRET (donor ex. 488, acceptor em. 630–800 nm, gain 900) to visualize GQ‐DNA, which was presented in red. The biofilms were visualized by CLSM (Zeiss LSM700) using 63× (for *S. oneidensis* monospecies) and 40× (for *B. subtilis* mono‐ and multispecies biofilms) oil‐immersed objectives.

Additionally, *B. subtilis* + *S. oneidensis* S2391 cocultured biofilms were visualized by single‐photon counting confocal fluorescence microscopy (Luminosa). TOTO‐1 (donor) was excited using the 488 nm laser (LDH‐D‐C‐485S, 40 nW – 9 µW) and detected around 500–540 nm (BP520/35). The FRET signal from SYTO60 was measured upon excitation with the 488 nm laser and detected around 650–725 nm (BP690/70). The direct SYTO60 signal (acceptor) was excited using the 640 nm laser (LDH‐D‐C‐640S, around 0.5 nW – 0.1 µW) and detected using the same BP690/70 filter. The 640 nm laser was always set to be 80x lower compared to the 488 nm laser. In several experiments, the *tn7*::CFP signal was also measured using the 405 nm laser (LDH‐D‐C‐405S, around 9 µW) and detected around 430–460 nm (BP445/30). For all pulsed lasers, a repetition rate of 20 MHz was used in pulsed interleaved excitation mode. The donor and FRET channels were kept at the same contrast range (for example, 0–50 counts), whereas the acceptor channel had half of this contrast range (for example, 0–25 counts). The CFP channel was kept at the same contrast setting as for the acceptor channel.

### 4.12 End‐Point Detection of Rolling Circle Replication in Biofilms Using cDNA and TSA

Biofilms were labeled with the cDNA oligonucleotide complementary to the products of DNA rolling circle replication (Table 1). We used universal cDNA (5 µM), unlabeled as well as labeled with 5’‐Alexa647, to tag products of RCR generated from various templates. Additionally, we used TSA as a method to specifically detect peroxidase activity of GQ‐DNAzymes in the presence of hydrogen peroxide. To detect such activity, the biofilms were obtained in TSB media doped with 20 µM hemin (Sigma; Porcine BioXtra; Cat.No. 51280) and 20 µM FeSO_4_. Subsequently, the media was replaced by Tris‐Acetate buffer (pH 6) with fluorescent Alexa488‐tyramide reagent (B40953, Thermo Fisher Scientific) diluted 1:100, 0.5 mM ATP, 0.2% H_2_O_2_, and 100 mM KCl, in which TSA was performed for around 2 h. The biofilms were visualized by CLSM (Zeiss LSM700) using 40x oil‐immersed objective. The cDNA and the tyramide fluorescence were shown in blue and green, respectively.

### 4.13 End‐Point Detection of Rolling Circle Replication in Biofilms Using Scanning Electron Microscopy

Scanning electron microscopy (SEM) analysis was performed on biofilms grown in μ‐Slide 8 well IbiTreat slides (Ibidi; Cat.No. 80826). The biofilms were gently rinsed with deionized water, decanted, and dried under vacuum overnight before sputter coating with a thin layer of platinum. The SEM images were acquired using a TESCAN Clara SEM under high vacuum (pressure <0.04 Pa), an accelerating voltage of 5 keV, a beam current of 100 pA and an Everhart‐Thornley (E‐T) detector for secondary electrons. SEM energy‐dispersive x‐ray spectroscopy (EDX) mapping was performed using a beam current of 3 nA.

### 4.14 Measurements of Redox Activity of eDNA Superstructures in Biofilms

Bioelectrochemical analyses of the monocultured *S. oneidensis* biofilms grown aerobically on screen‐printed electrodes (SPEs) were carried out on the SPEs (Metrohm DropSens DRP‐C110, Metrohm Spain). It had a 4 mm diameter graphite working electrode (WE) with a surface area of 0.126 cm^2^, a graphite counter electrode, and Ag pseudo‐reference electrode. SPEs were surface sterilized in 70% v/v ethanol, washed thrice in sterile deionized water, air dried, under aseptic conditions and exposed to UV treatment for 15 min. Biofilms were grown for 3 days on SPEs in 1 mL glass tubes with butyl rubber caps fused to WE portion of SPEs prior to analyses [3]. Biofilms were grown 3‐days aerobically (30°C, 100 rpm) and additionally 3‐days anaerobically (30°C, 100 rpm). After incubation and biofilm formation, SPEs with formed biofilms were immediately connected to a PC‐controlled multichannel PalmSens4 potentiostat (PalmSens Netherlands). Differential Pulse Voltammetry (DPV) analyses were carried out using electrode potential spanning from ‐0.8 V to 1.5 V and following parameters: 5 s equilibration, 0.01 V step, 0.05 V/ 0.3 s pulse and 5 mV/s scan rate. Two biological replicates of *S. oneidensis* were prepared for each test condition, and three technical replicates (measurements) for each sample were recorded and averaged. All analyses were carried out in open laboratory conditions and with no disruption of sample.

## Author Contributions

**Gabriel Antonio S. Minero**: conceptualization, investigation, funding acquisition, writing – original draft, methodology, validation, visualization, writing – review and editing, project administration, supervision, resources. **Line Mørkholt Lund**: validation, visualization, methodology, writing – review and editing, investigation. **Lasse Hyldgaard Klausen**: methodology, validation, visualization, writing – review and editing, investigation. **Obinna Markraphael Ajunwa**: visualization, validation, methodology, writing – review and editing, investigation. **Mingdong Dong**: methodology, supervision, validation. **Rikke Louise Meyer**: resources, methodology, writing – review and editing, validation. **Victoria Birkedal**: methodology, writing – review and editing, resources, validation, supervision. **Kai Thormann**: writing – review and editing, methodology, validation, conceptualization.

## Funding

We thank the Villum Foundation, grant no. 00050284 for funding a major part of this work and grant 00069632 for finalizing publication of this work. We thank the Danish council for independent research, grant no. 2032‐00294B, for funding the biofilm electroactivity tests. We gratefully acknowledge the support and use of the Aarhus single molecule fluorescence (ASiMoF) infrastructure, funded by the Novo Nordisk Foundation (NNF20OC0061417), for the fluorescence imaging of biofilms with single photon counting confocal microscopy. We also acknowledge funding from the Carlsberg Foundation (Grant no. CF20‐0364), the Horizon Europe Marie Skłodowska‐Curie Actions (project 101086226‐ENSIGN) and Center for Integrated Materials Research (Aarhus University) for the biofilm SEM tests.

## Conflicts of Interest

The authors declare no conflicts of interest.

## Supporting information




**Supporting File**: anie74064‐sup‐0001‐SuppMat.pdf.

## Data Availability

The data that support the findings of this study are available from the corresponding author upon reasonable request.
