# Supplementary material for: Prophage Activation Triggers Extracellular Synthesis of DNA in Bacterial Biofilms via Rolling Circle Replication
Source: Angew Chem Int Ed Engl. 2026 Aug 24;65(41):e3695434. doi: 10.1002/anie.3695434 (PMC13638115; doi:10.1002/anie.3695434)
Supplement: Supplementary file 1 [file ANIE-65-e3695434-s001.pdf]

Prophage Activation Triggers Extracellular Synthesis of DNA in Bacterial Biofilms via Rolling Circle Replication

Gabriel Antonio S. Minero *et al.* (antonio.minero@inano.au.dk, minero.gabriel@hotmail.com)

ELECTRONIC SUPPLEMENTARY INFORMATION

Table S1: Summary of all the experiments and controls used in this work to detect the extracellular template-directed synthesis of DNA. In the left column, the bacterial strains and the DNA templates are shown. In the upper raw, various fluorescent and elemental analyses are shown. Each Figure used in this work can be identified by the experimental conditions listed in this Table.

	Fluorescent exogeneous circular DNA	NMP	TOTO™-1 + SYTO™60	cDNA	Tyramide signal amplification	SEM-EDX
**T1**		Figure S1				
S. oneidensis WT **+ T1**		Figures 2A, 3C	Figures 3B, 4A, S5-S7	Figure S5		Figures 3F, 4B-D
S. oneidensis WT (*cro*::Venus) **+ T1**		Figures 1B,C				
S. oneidensis WT **+** Atto405-**T2**	Figure 2D					
S. oneidensis WT			Figure S6			
S. oneidensis MUT **+ T1**			Figure S7			
B. subtilis WT + **T1**	Figure 2C			Figure 5B Figure S8B	Figure 5A Figure S8A	Figure S8C,D
B. subtilis WT + Atto405-**T2**	Figures 2D, S3					
S. oneidensis / *B. subtilis* (WT/WT) + **T1**				Figure 6A	Figure 6A	
S. oneidensis (*cro*::Venus) /*B. subtilis* (WT) + **T1**		Figure S10	Figure S9			
S. oneidensis / *B. subtilis* (WT/WT) + **T2**				Figure 6B	Figure 6B	
S. oneidensis / *B. subtilis* (WT/WT)				Figure 6C	Figure 6C	

Cell-free rolling circle replication of GQ-DNA from T1 circular DNA template detected by NMP difference readout

Rolling Circle Replication (RCR) is a well-established technique to replicate a circular DNA using phage polymerases with strand-displacing activity. Figure S1 shows increase of "NMP difference" in RCR templated by the ligated circular DNA (25-200 nM). In this technique, a "padlock probe" or template (shown in blue), 5'-phosphorilated, is first annealed and ligated onto a single-stranded DNA "primer" using Ampligase or another DNA ligase. In the presence of nucleotide triphosphates (NTPs) and polymerase, the 3'-end of the primer (shown in red) is extended into the growing DNA chain, resulting in release of pyrophosphates. After the full circle of DNA template is completed, polymerase uses its strand displacing activity to keep rolling circle replication until all NTPs are depleted or polymerase is inactivated. We used T1 circular template containing triplets of cytosines to generate triplets of guanines foldable into G-quadruplex GQ-DNA as the end-product of RCR in the presence of commercial phi29 polymerase. In the presence of the synthesized GQ-DNA, the N-Methyl Protoporphyrin (NMP) fluorescence lights up that makes it possible to detect such product of rolling circle replication. We deducted NMP fluorescence of non-templated controls from the samples templated with 25 nM, 100 nM and 200 nM circular DNA and show the dose-response signal saturating in all samples around 120 min suggesting inactivation of the phi29 polymerase.

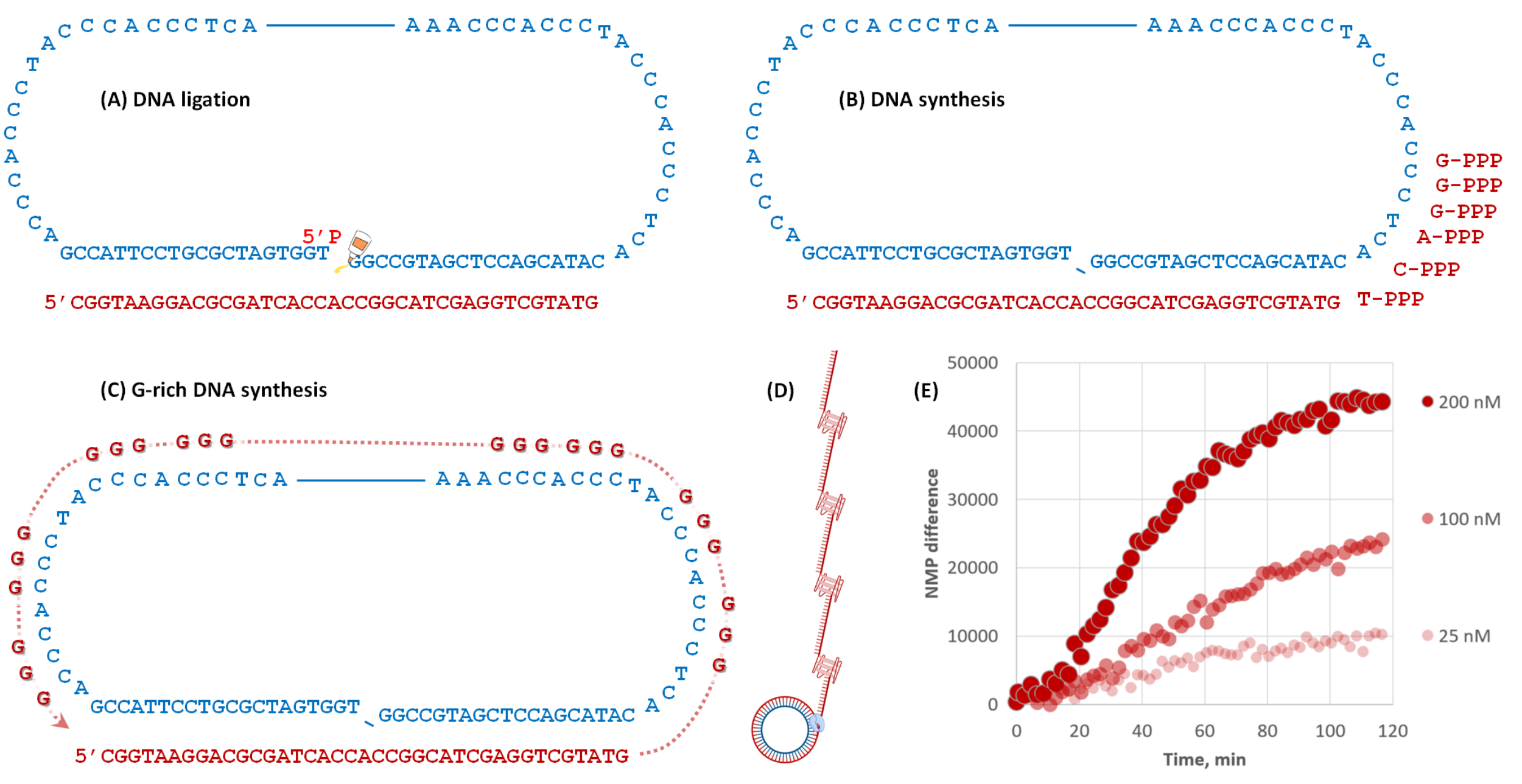

Figure S1. Real-time detection of rolling circle replication of GQ-DNA from template T1 using phi29 polymerase, dNTP and different concentrations of the circular DNA T1. (A) Ligation the circularized DNA template is followed by (B) Incorporation of NTPs, DNA synthesis and, ultimately (C) rolling circle replication resulting in an end-product containing desired sequence, e.g. (D) GQ-motifs (synthesized using T1 template). (E) Detection of the RCR using "NMP difference".

One turn around T1 template resulted in eight triplets of guanines foldable into two GQs separated by two DNA tracks (the conservative sequences in T1 and T2 are highlighted in grey):

(-CGGTAAGGACGCGATCACCACCGGCATCGAGGTCGTATGTGA**GGG**T**GGG**TA**GGG**T**GGG**TTTGTGGCAGGTCAGTCAAGTATACTGCACTATTTGA**GGG**T**GGG**TA**GGG**T**GGG**T-)n.

Rolling Circle Replication is inhibited in the extracellular space of *Shewanella oneidensis* in the presence of hydrogen peroxide

Real-time monitoring of a “negative” GQ-DNA signal calculated via NMP difference (non-templated control fluorescence being subtracted) despite active phage proliferation (Figure S2) detected via the fluorescent proteins expressed by *S. oneidensis* (*tn7*::CFP in violet and *cro*::Venus in green).

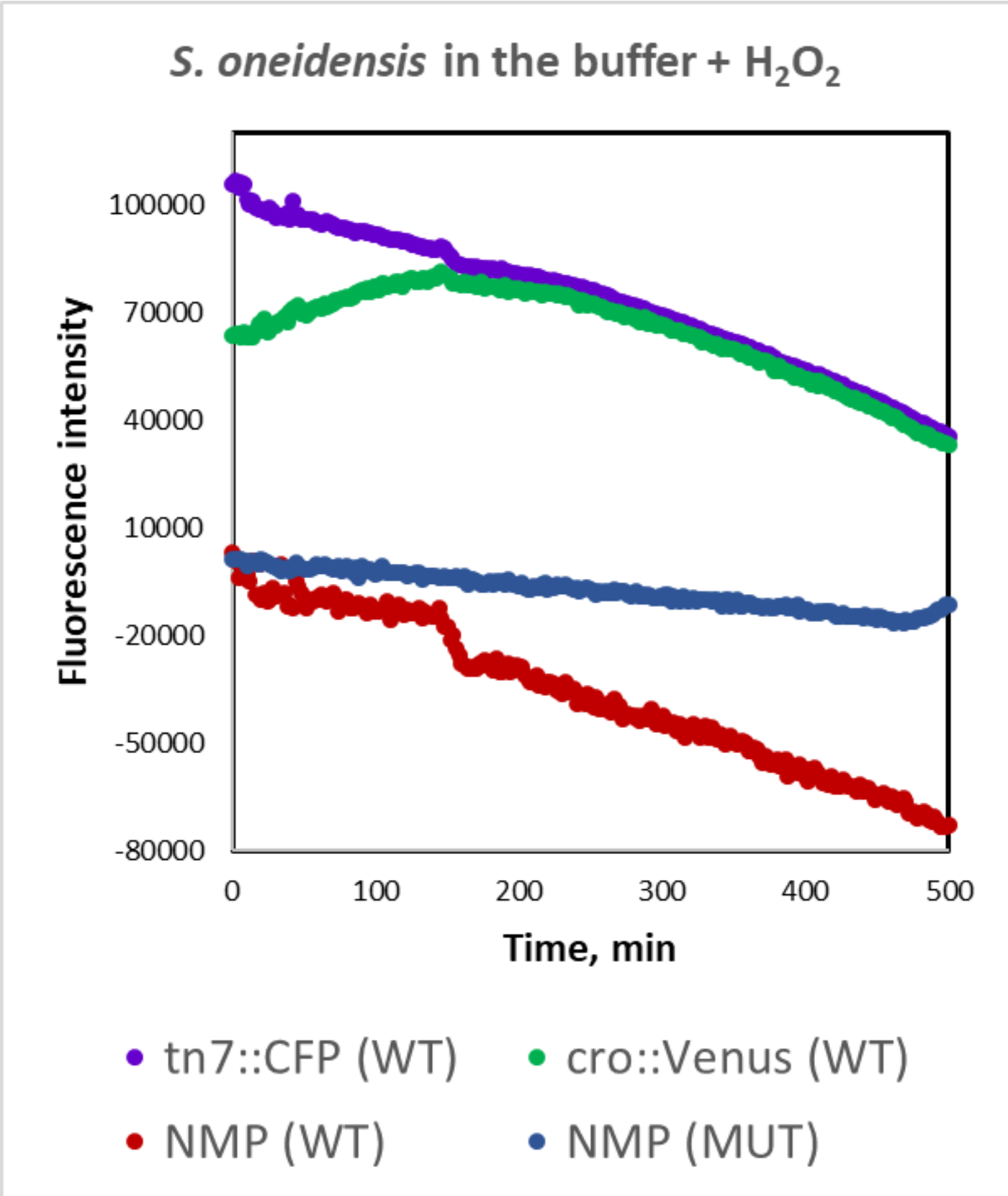

Figure S2: Monitoring *Shewanella oneidensis* reporter strain (S2391, *tn7*::CFP, *cro*::Venus) under nutrient-limited conditions in the presence of 0.2% H2O2 in real time. As described in Figure 1, prophage activity leads to the cells are being lysed (violet curve), the prophage lambda is being actively replicated (green curve), and the NMP difference is measured in the wild-type (+ phage) and mutant (ΔλSo ΔMuSoI ΔMuSoII) cultures (in red and blue, respectively) in the presence of 150 nM T1 circular template. To activate phage replication in the wild-type *S. oneidensis* reporter strain, we also used mitomycin C (Figure 1B), when we detected clear increasing NMP fluorescence.

Detection of DNA wire-like superstructures in *B. subtilis* planktonic cultures supplemented with T2 circular DNA template for rolling circle replication of R-DNA

We deliberately chose low GC scrambled DNA template T2 to demonstrate that the superstructure formation is not necessarily linked to G-quadruplex, but any concatemer DNA sequence such as the one generated from T2 template can form the wire-like superstructures in the extracellular space of *B. subtilis* (Figure S3).

One turn around T2 template resulted in the following sequence (the conservative sequences in T1 and T2 are highlighted in grey):

5'(-CGGTAAGGACGCGATCACCACCGGCATCGAGGTCGTATGTAAGACACTATTACGTGGCAGGTCAGTCAAGTATACTGCACTAATGCTC**GGG**AA**GG**CTACT -)n.

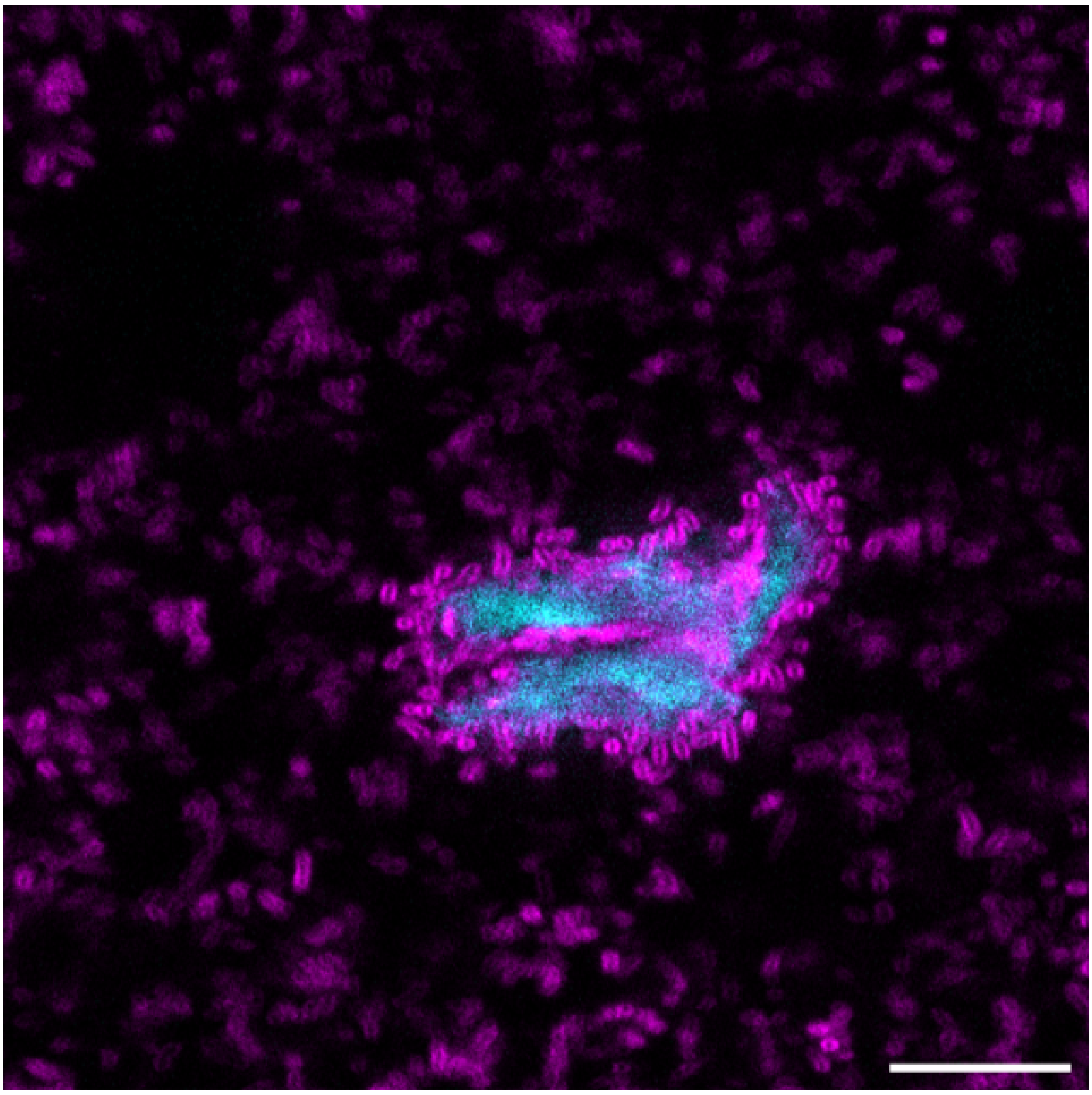

Figure S3: Fluorescence imaging of *B. subtilis* with single photon counting confocal microscopy of FM 4-64 lipophilic membrane stain (in magenta) and fluorescently labeled circular DNA T2 (in cyan) detected 360 min of continuous incubation of bacterial cells with DNA. Scale bar 10 µm. See Figure 2 for zoomed in images at different time points of incubation.

SYTO™60/ TOTO™-1 FRET pair detects GQ-DNA and does not detect scrambled polyGC DNA

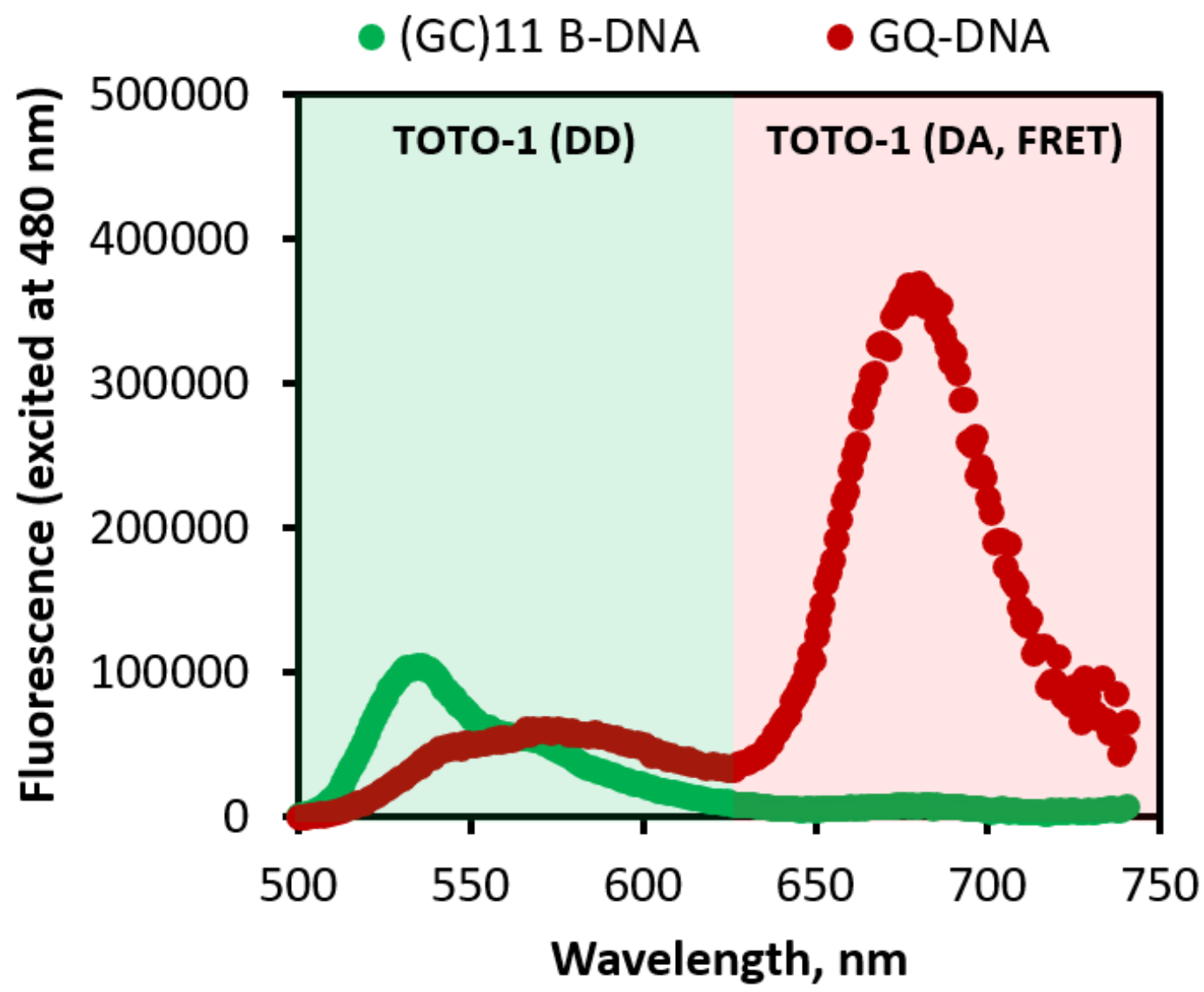

Figure S4: TOTO™-1 and SYTO™60 staining of DNA results in FRET signal highly specific for GQ-DNA. Comparison of the FRET signal in synthetic GQ-DNA vs. scrambled (GC)11 B-DNA in Tris buffer with 100 mM KCl. Excitation wavelength was 480 nm, and same gain settings were applied in the CLARIOstar plate reader.

We tested our novel FRET-based detection of GQ-DNA using SYTO™60 and TOTO™-1 dyes in the presence of the GQ-DNA (GA**GGG**T**GGG**TA**GGG**T**GGG**) and scrambled G-rich B-DNA (GCGCGCGCGCGCGCGCGCGCGC). The TOTO™-1 signal was clearly shifted from the green to the red part of the fluorescence spectrum due to FRET solely in GQ-DNA (unlike G-rich B-DNA) when annealed in the plain potassium-rich buffer (25 mM Tris-Acetate, pH 7, with 100 mM KCl).

CLSM imaging of T1-templated *S. oneidensis* biofilms using SYTO™60/ TOTO™-1 FRET pair detects GQ-DNA within spherical superstructures

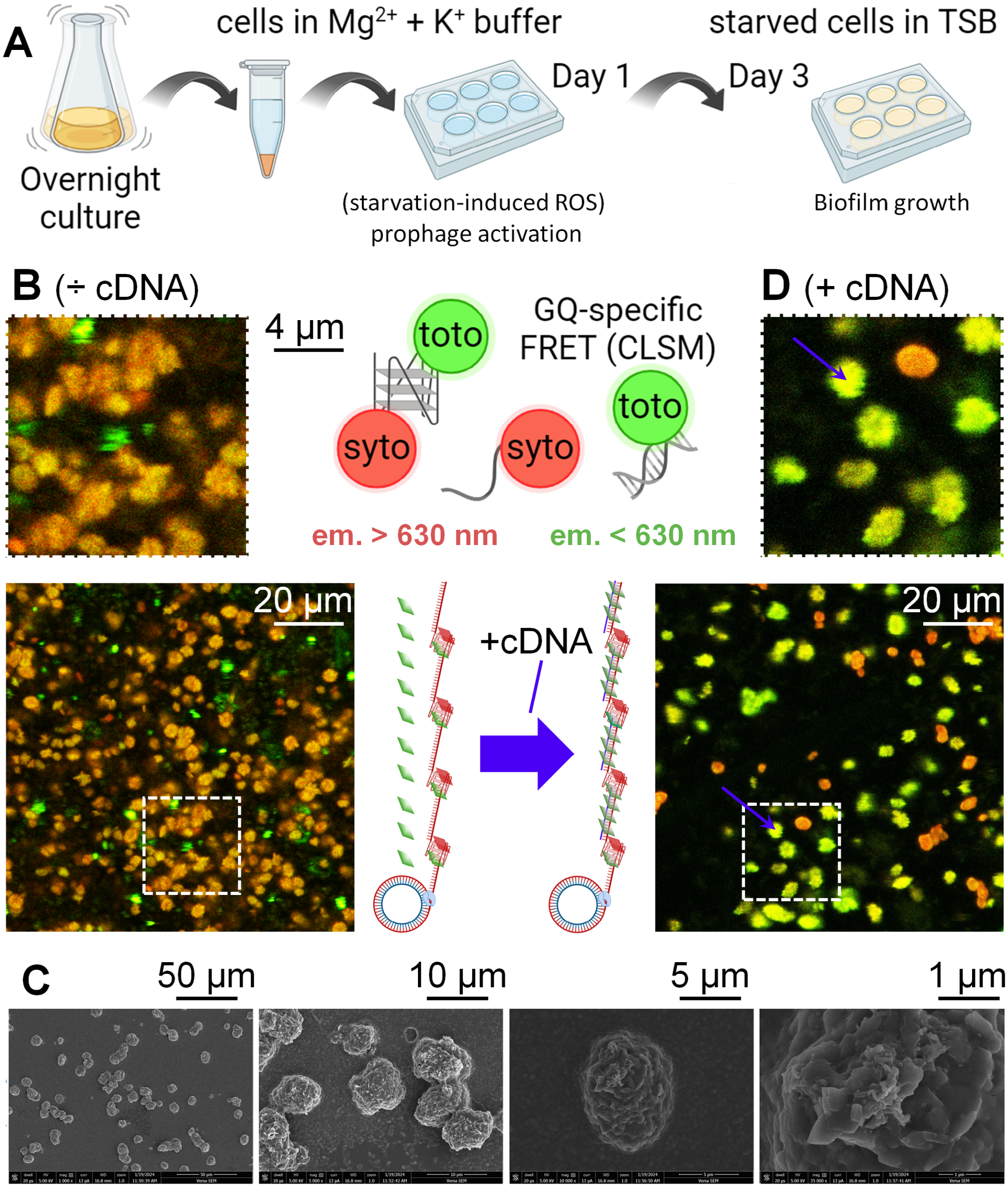

Figure S5. CLSM of GQ-DNA superstructures replicated from template T1 in 3-day biofilms of wild-type *S. oneidensis* stained with SYTO™60/TOTO™-1. (A) The workflow used for biofilm formation with starvation-induced RCR (shown by black arrows). (B) Upon biofilm staining, both TOTO™-1 and SYTO™60 bind to GQ-DNA and cause FRET. High FRET signal (red) detected in spherical structures indicates the end-product of RCR enriched in the multiple GQ motifs. Cartoons of GQ-, single- and double-stranded DNA existing in the extracellular matrix and interacting with the dyes. (C) Replication of the circular DNA T1 in *S. oneidensis* biofilms leads to formation of crystal-like spherical super-structures detected by SEM. (D) The RCR end-product is complementary to cDNA. High TOTO™-1 signal (green) detected in spherical structures indicates an increased amount of dsDNA between cDNA oligonucleotide and the end-product of RCR upon addition of 10 µM cDNA (shown by blue arrow). TOTO™-1 and SYTO™60 are represented by green and red cartoons, respectively.

Figure S5 shows RCR assay first in bacterial cultures under nutrient-limiting conditions (1-day) and then in biofilms grown for 2-days in TSB media. We, furthermore, used live/ dead staining by SYTO™60/ TOTO™-1 DNA-binding dyes to detect synthesized eDNA superstructures. TOTO™-1 normally lights up B-DNA and dead cells (green) and does not light up GQ-DNA. We recently established that GQ motifs bind SYTO™60 and TOTO™-1 resulting in Förster Resonance Energy Transfer (FRET) (*Lund et al., Nucl. Acids Res. (2026) gkag712, accepted*). To detect GQ-DNA, SYTO™60 (acceptor) emission (> 630 nm) is detected upon TOTO™-1 (donor) excitation (488 nm) *that we call donor/acceptor (DA)*. To simultaneously detect B-DNA, TOTO™-1 (donor) emission (<630 nm) is detected upon TOTO™-1 (donor) excitation (488 nm) *that we call donor/donor (DD)*. In the extracellular space, we found that TOTO™-1 and SYTO™60 form a FRET pair resulting in SYTO™60 (acceptor) emission (red) upon TOTO™-1 (donor) excitation (Figure S5B). The morphology of the spherical structures forming in the DNA templated *S. oneidensis* WT biofilms was furthermore visualized by SEM (Figure S5C). To simultaneously detect cells, SYTO™60 (acceptor) emission (> 630 nm) is detected upon SYTO™60 (acceptor) excitation (639 nm) *that we call acceptor/acceptor (AA).*

Furthermore, we labeled biofilms with cDNA probe (Table 1) forming double-stranded B-DNA with the single-stranded product of RCR to confirm sequence of the RCR product (shown by blue arrow in Figure S5). Using the two DNA-binding dyes allowed us to detect cDNA binding to the DNA products synthesized from template T1. After 1 h incubation of the spherical superstructures (in *S. oneidensis* biofilm, Figure S5B) with the cDNA, a sub-population of the spherical structures increased in TOTO™-1 fluorescence (emission < 630 nm, shown in green), indicating formation of double-stranded B-DNA (Figure S5D). However, another sub-population of spherical structures did not light up TOTO™-1 (did not become more "green") after cDNA labeling, indicating a barrier to the cDNA probe or the TOTO™-1 dye. These could be, for example, protoplasts with a remaining lipid barrier preventing passage of the cDNA and are distinct from another sub-population of the spherical structures. Therefore, we assumed that there are two phenotypes of biofilms with the RCR induced in (1) the extracellular or (2) the intracellular environment. In contrast to planktonic assay of < 24 h, uptake of the circular DNA templates is, therefore, possible during 3-day biofilm formation.

No FRET as well as no spherical superstructures were detected in the non-templated WT biofilms (Figure S6) or templated *S. oneidensis* MUT (ΔλSo ΔMuSoI ΔMuSoII) biofilms (Figure S7).

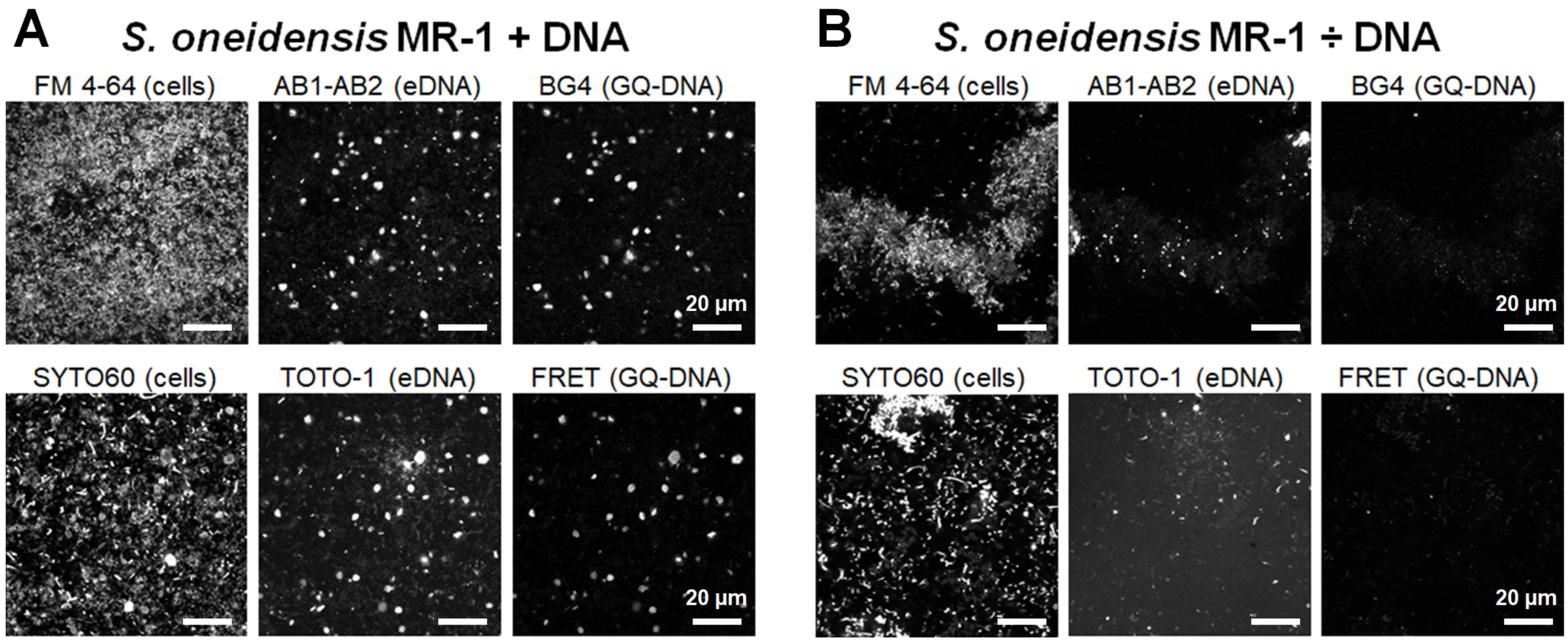

Figure S6. ***S. oneidensis*** **biofilms doped with circular DNA template T1 form spherical superstructures detected by the GQ-specific immunolabeling and the FRET pair.** (A) Biofilms obtained in DNA-TSB-NaCl containing 200 nM exogenous circular DNA template and (B) biofilms obtained in plain TSB-NaCl were labeled by DNA-specific antibodies BG4 and AB1-AB2 in combination with FM 4-64, as well as by DNA-binding stains TOTO™-1 and SYTO™60. Scale bars 20 µm.

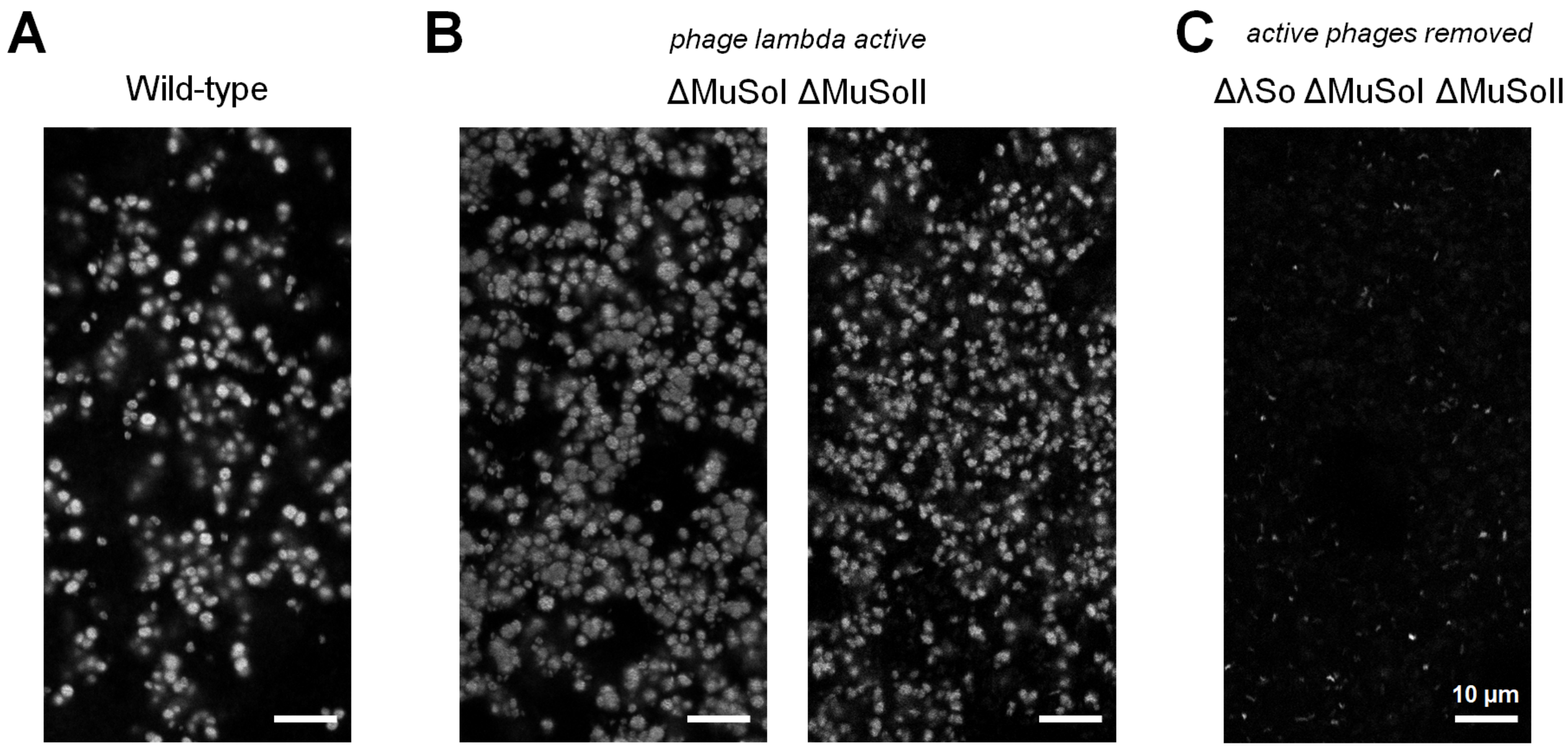

Figure S7. Prophage λSo proliferation is responsible for extracellular synthesis of GQ-DNA in *S. oneidensis* 3-day old biofilms. CLSM (FRET channel) of the replicated DNA template T1 in 3-day biofilms of *S. oneidensis* MR-1 strains: (A) WT, (B) ΔMuSoI ΔMuSoII and (C) ΔλSo ΔMuSoI ΔMuSoII stained with TOTO™-1 and SYTO™60. Scale bars 10 µm.

Analysis of T1-templated *B. subtilis* biofilm producing wire-like superstructures

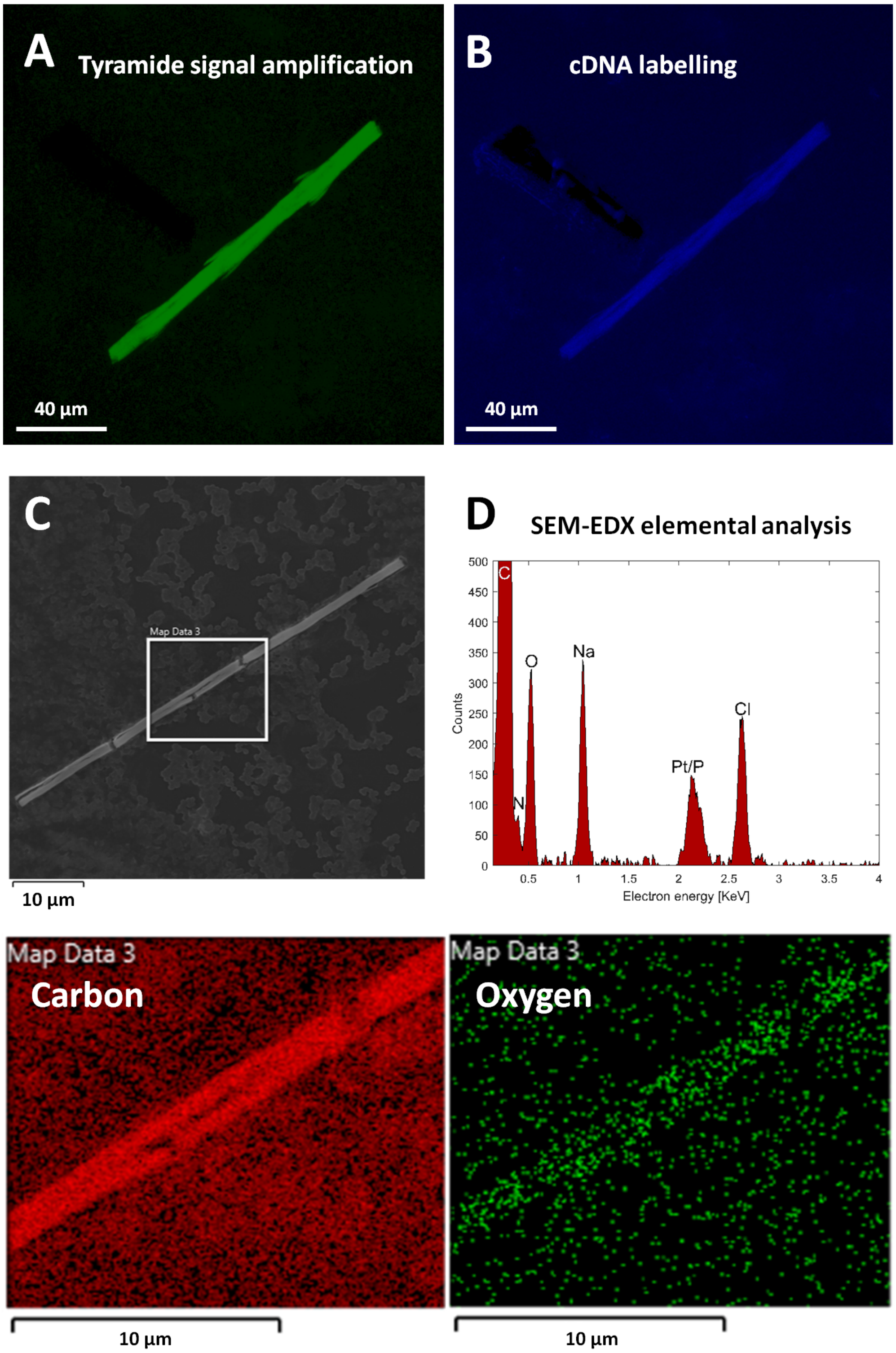

Figure S8. (A-B) Typical wire-like superstructures detected in 3-day *B. subtilis* biofilm and visualized using fluorescence CLSM maximum intensity projection after tyramide signal amplification and cDNA labelling. Scale bars 40 µm. **(C-D) SEM-EDX elemental analysis of the wire-like superstructures indicative of high contents of carbon and oxygen.** Scale bars 10 µm.

Fluorescence imaging of T1-templated *S. oneidensis* + *B. subtilis* biofilms using SYTO™60/ TOTO™-1 FRET pair detects GQ-DNA within wire-like superstructures

Next, we wondered if formation of the wire-like superstructures detected in *B. subtilis* biofilms is influenced by *S. oneidensis* when co-cultured with *B. subtilis.* We used the same assay and workflow as in Figure S5 and the S2391 reporter strain of *S. oneidensis* constituently expressing CFP protein from its tn7 promoter. The biofilms were stained with TOTO™-1 and SYTO™60 couple resulting in FRET signal specific for GQ-DNA. We noticed very high FRET signal (red) from a wire-like structure close to 1 mm in length (Figure S9). Furthermore, we detected the CFP produced from *S. oneidensis* S2391 (*tn7*::CFP) within the wire (Figure S9B) suggesting that CFP among other biopolymers derived from *S. oneidensis* contributes to the wire-like superstructure typically found in *B. subtilis* (in this work).

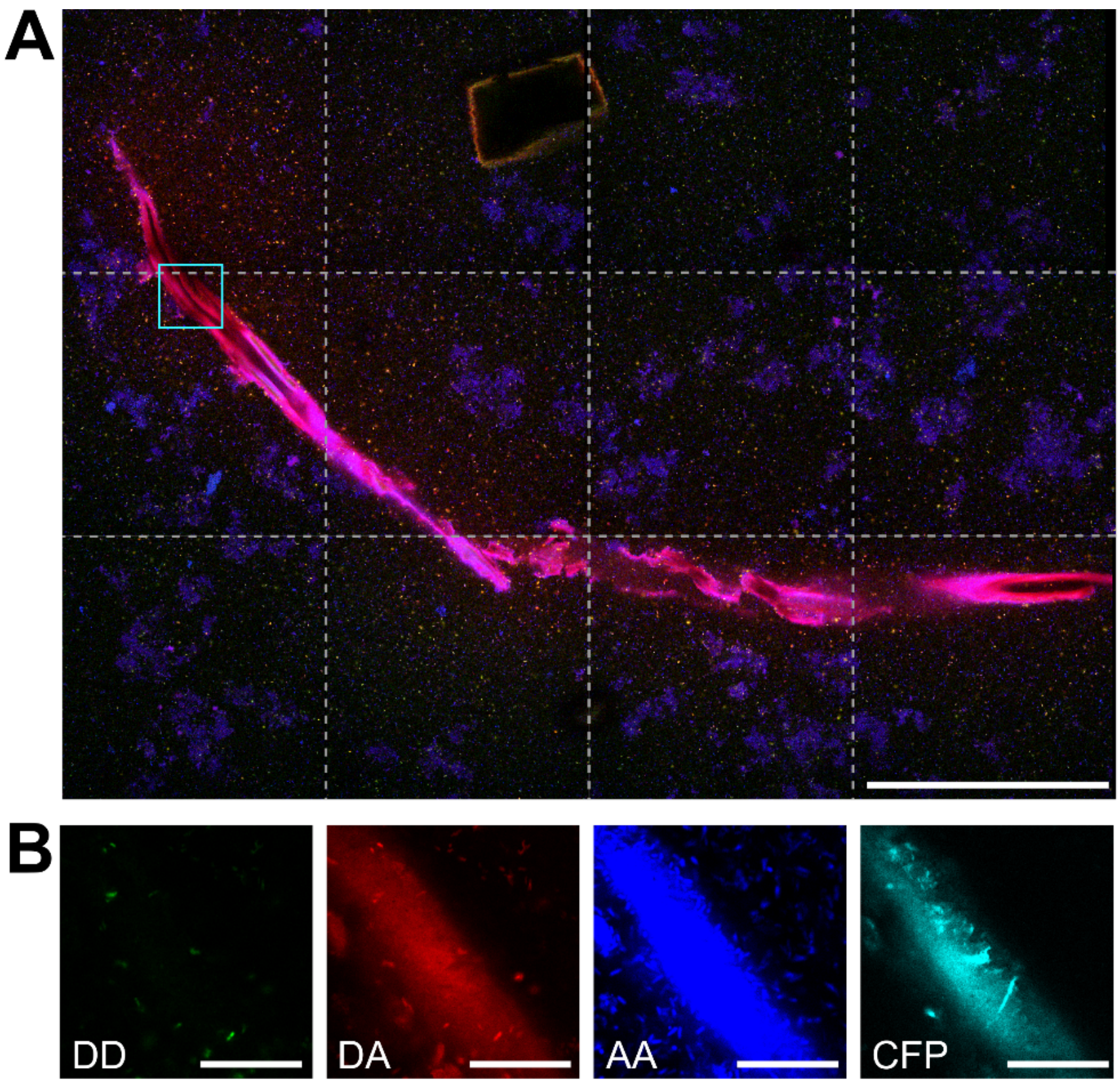

Figure S9. FRET in GQ-DNA construct produced in *S. oneidensis* and *B. subtilis* (co-cultured) biofilm (day 3) in TSB-KCl detected by single photon counting confocal microscopy. (A) Example of a millimeter long, twisting wire. Here, green is from the donor (DD, TOTO™-1 channel), red is from FRET signal (DA, SYTO™60 channel), and blue is from the directly excited acceptor (AA, SYTO™60 channel). Since the difference in detection efficiency of green and red signal is 1:2.5, the signal in the green channel has been multiplied by 2.5. Still, the wire is red overall, meaning high in the FRET signal (DA). Scale bar is 200 µm. (B) Small cutout of the same wire in (A), cyan box, at a different z-height to show the surface of the wire. Here, the fluorescent signal is split into four channels. The donor signal in green (DD, TOTO™-1 channel), the FRET signal in red (DA, SYTO™60 channel), direct acceptor in blue (AA, SYTO™60 signal), and *tn7*::CFP signal in cyan (CFP channel). Scale bars are 20 µm.

The CFP expressed specifically by *S. oneidensis* S2391 was found to be present in biofilms of monospecies S2391 (Figure S10A) as well as co-cultured with *B. subtilis* (Figures S9, S10C). Therefore, we believe that the pathways of DNA replication resulting in the giant DNA wires are similar in *B. subtilis* and *S. oneidensis*.

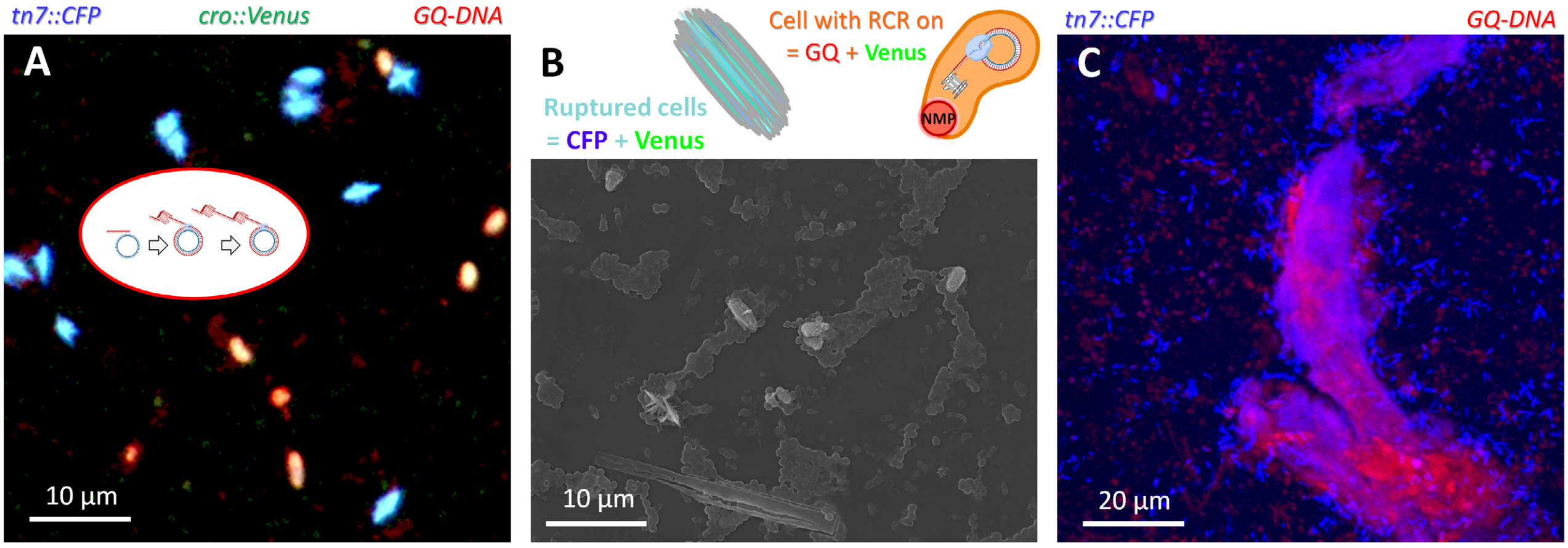

Figure S10. CFP from *S. oneidensis* is deposited into the supramolecular structures synthesized and self-assembled in 3-day biofilms of *S. oneidensis* S2391 (*cro*::Venus, *tn7*::CFP) co-cultured with *B. subtilis*. (A) CLSM and (B) SEM images of cells being ruptured. NMP signal predominantly detected inside deformed cells of *S. oneidensis* with active synthesis of *cro* (*cro*::GFP indicative of prophage λSo proliferation, green). Ruptured cells show high signal of both CFP (blue) and Venus (green) proteins. (C) The wire-like superstructure detected by NMP (indicative of the GQ end-product of RCR) and CFP (indicative of *S. oneidensis*) signals.

Fluorescence imaging of *Staphylococcus epidermidis* biofilms using SYTO™60/ TOTO™-1 detects wire-like superstructures

Staphylococcus epidermidis (strain 1457) biofilms were inoculated in TSB-NaCl from overnight cultures (OD 0.1) and were grown in TSB-NaCl for 10-days. The obtained 10-day biofilms were stained in 12.5 µM SYTO™60 and 2.5 µM TOTO™-1 solution in 100 mM NaCl and visualized in LSM900 Zeiss microscope using 488 nm laser excitation / collecting emission < 590 nm (DD, green) and > 630 nm (DA, FRET, red) and using 640 nm laser/ collecting emission > 630 nm (AA, blue). Figure S11 shows formation of wire-like superstructures stained by these DNA-binding dyes.

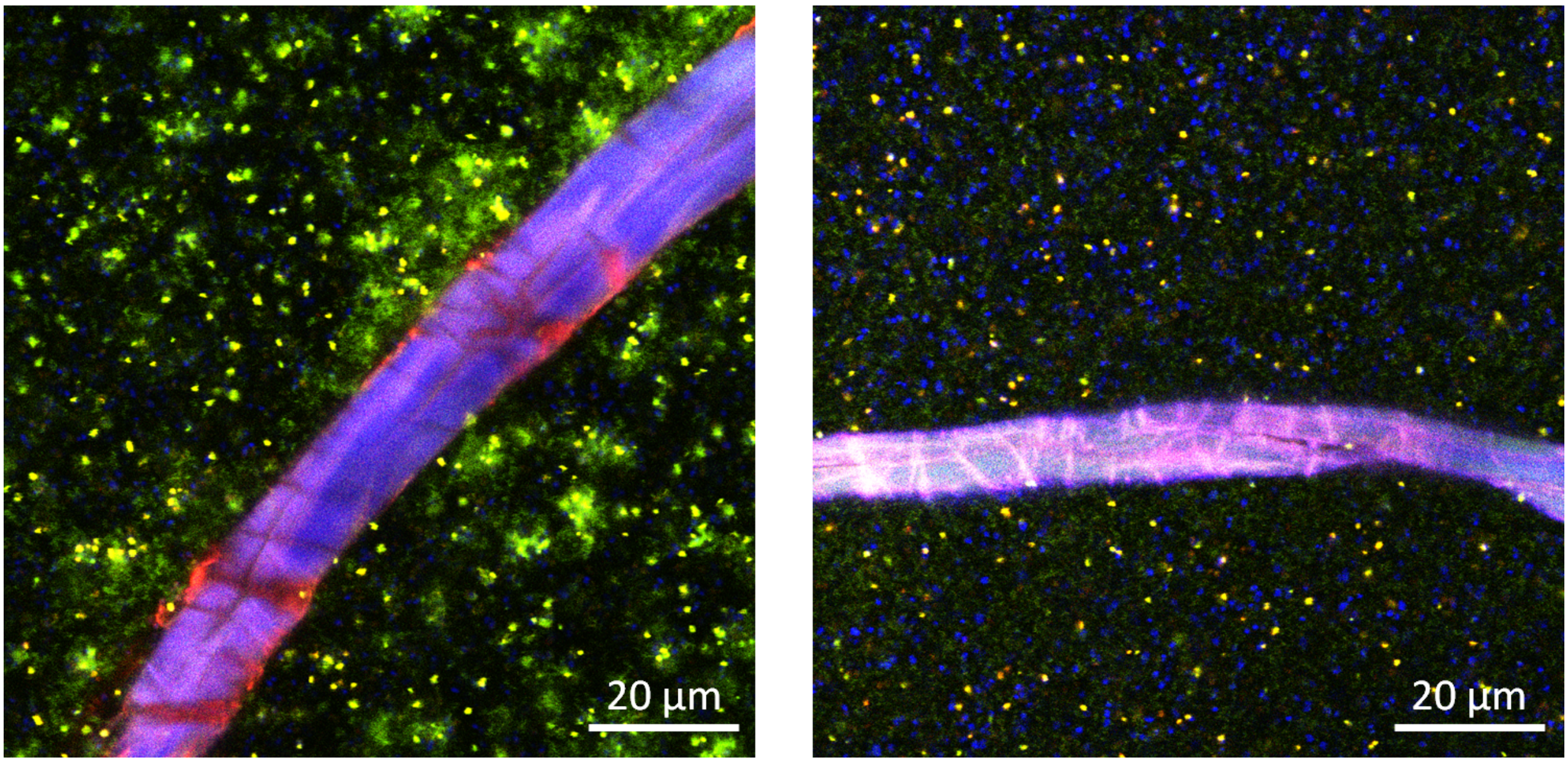

Figure S11. Formation of wire-like superstructures in 10-days *S. epidermidis* biofilms detected by SYTO™60/ TOTO™-1 FRET pair. SYTO™60 signal (DNA, live cells) is shown in blue. TOTO™-1 signal is shown in green (DNA, dead cells) and red (FRET = GQ-DNA). Two biological replicates of *S. epidermidis* biofilms are shown. Scale bars 20 µm.

49 Spadina Ave. Suite 200
Toronto ON M5V 2J1 Canada
www.biorender.com

Confirmation of Publication and Licensing Rights

August 9th, 2026

Subscription Type:	*Institution - Academic*
Agreement number:	*EV2A27IVO3*
Publisher Name:	*Angewandte Chemie*
Citation to Use:	*Created in BioRender. Meyer, R. (2026) https://BioRender.com/68plb9t*

To whom this may concern,

This document is to confirm that Rikke Meyer has been granted a license to use the BioRender Content, including icons, templates, and other original artwork, appearing in the attached Completed Graphic pursuant to BioRender's Academic License Terms. This license permits BioRender Content to be sublicensed for use in publications (journals, textbooks, websites, etc.).

All rights and ownership of BioRender Content are reserved by BioRender. All Completed Graphics must be accompanied by the following citation: "Created in BioRender. Meyer, R. (2026) https://BioRender.com/68plb9t".

BioRender Content included in the Completed Graphic is not licensed for any commercial uses beyond use in a publication. For any commercial use of this figure, users may, if allowed, recreate it in BioRender under an Industry BioRender Plan.

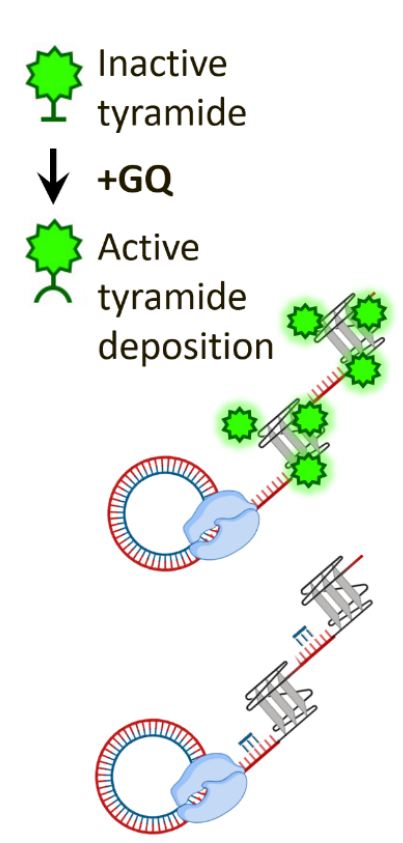

For any questions regarding this document, or other questions about publishing with BioRender, please refer to our BioRender Publication Guide, or contact BioRender Support at support@biorender.com.

49 Spadina Ave. Suite 200
Toronto ON M5V 2J1 Canada
www.biorender.com

Confirmation of Publication and Licensing Rights

August 9th, 2026

Subscription Type: *Institution - Academic*
Agreement number: *HB2A27JY28*
Publisher Name: *Angewandte chemie*

Citation to Use: *Created in BioRender. Meyer, R. (2026) https://BioRender.com/7v1oxcs*

To whom this may concern,

This document is to confirm that Rikke Meyer has been granted a license to use the BioRender Content, including icons, templates, and other original artwork, appearing in the attached Completed Graphic pursuant to BioRender's Academic License Terms. This license permits BioRender Content to be sublicensed for use in publications (journals, textbooks, websites, etc.).

All rights and ownership of BioRender Content are reserved by BioRender. All Completed Graphics must be accompanied by the following citation: "Created in BioRender. Meyer, R. (2026) https://BioRender.com/7v1oxcs".

BioRender Content included in the Completed Graphic is not licensed for any commercial uses beyond use in a publication. For any commercial use of this figure, users may, if allowed, recreate it in BioRender under an Industry BioRender Plan.

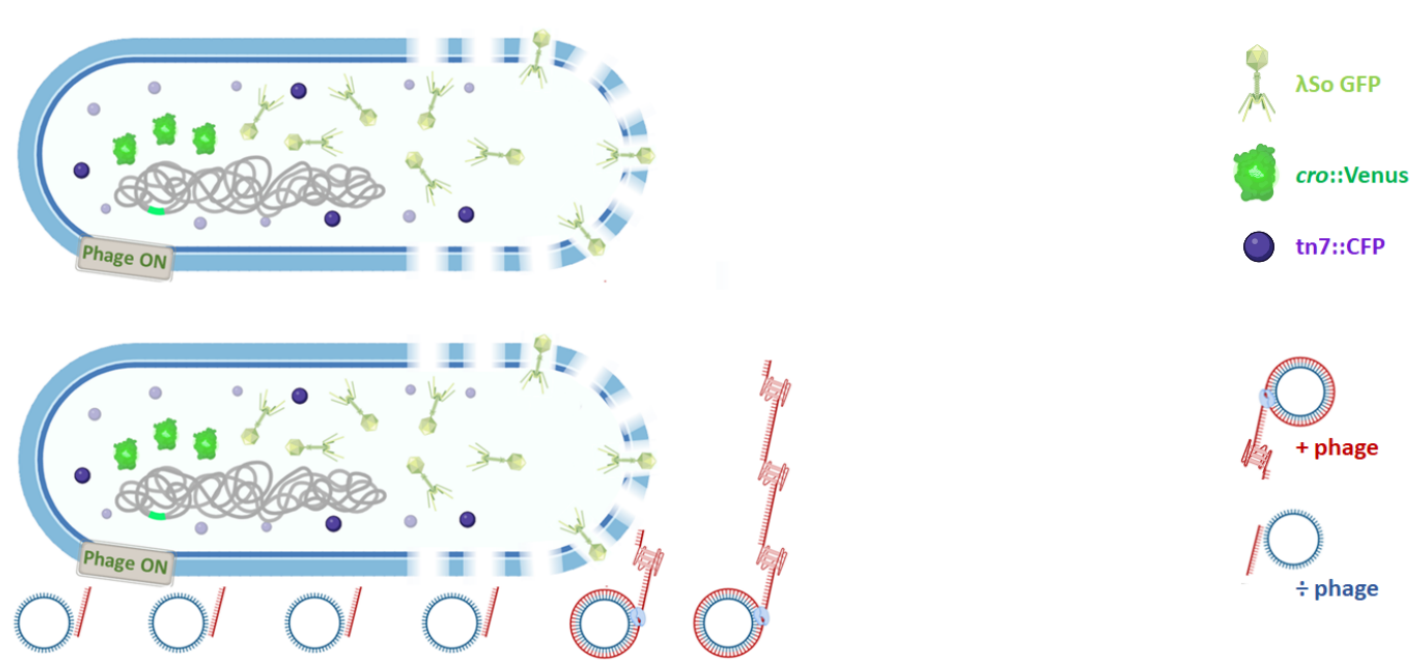

For any questions regarding this document, or other questions about publishing with BioRender, please refer to our BioRender Publication Guide, or contact BioRender Support at support@biorender.com.

49 Spadina Ave. Suite 200
Toronto ON M5V 2J1 Canada
www.biorender.com

Confirmation of Publication and Licensing Rights

August 9th, 2026

Subscription Type: *Institution - Academic*
Agreement number: *JV2A27J96C*
Publisher Name: *Angewandte Chemie*

Citation to Use: *Created in BioRender. Meyer, R. (2026) https://BioRender.com/bdjexuv*

To whom this may concern,

This document is to confirm that Rikke Meyer has been granted a license to use the BioRender Content, including icons, templates, and other original artwork, appearing in the attached Completed Graphic pursuant to BioRender's Academic License Terms. This license permits BioRender Content to be sublicensed for use in publications (journals, textbooks, websites, etc.).

All rights and ownership of BioRender Content are reserved by BioRender. All Completed Graphics must be accompanied by the following citation: "Created in BioRender. Meyer, R. (2026) https://BioRender.com/bdjexuv".

BioRender Content included in the Completed Graphic is not licensed for any commercial uses beyond use in a publication. For any commercial use of this figure, users may, if allowed, recreate it in BioRender under an Industry BioRender Plan.

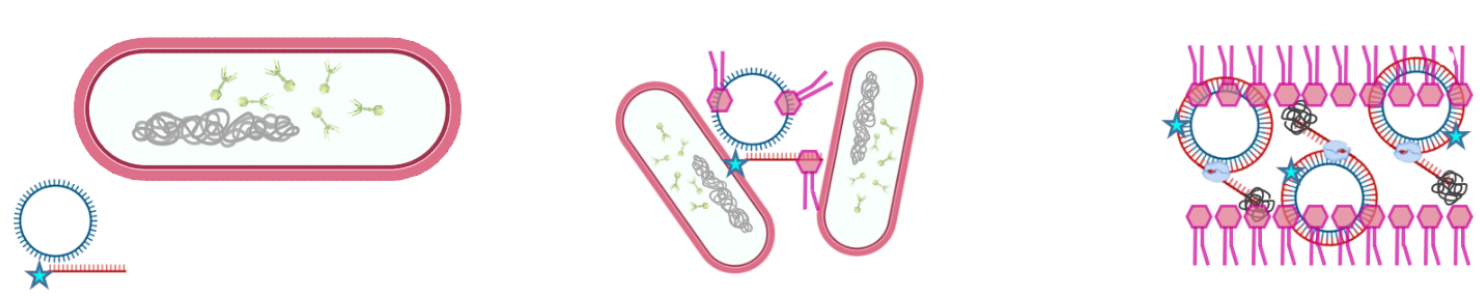

For any questions regarding this document, or other questions about publishing with BioRender, please refer to our BioRender Publication Guide, or contact BioRender Support at support@biorender.com.

49 Spadina Ave. Suite 200
Toronto ON M5V 2J1 Canada
www.biorender.com

Confirmation of Publication and Licensing Rights

August 9th, 2026

Subscription Type:	*Institution - Academic*
Agreement number:	*TJ2A27JEIX*
Publisher Name:	*Angewandte chemie*
Citation to Use:	*Created in BioRender. Meyer, R. (2026) https://BioRender.com/4s9xc6n*

To whom this may concern,

This document is to confirm that Rikke Meyer has been granted a license to use the BioRender Content, including icons, templates, and other original artwork, appearing in the attached Completed Graphic pursuant to BioRender's Academic License Terms. This license permits BioRender Content to be sublicensed for use in publications (journals, textbooks, websites, etc.).

All rights and ownership of BioRender Content are reserved by BioRender. All Completed Graphics must be accompanied by the following citation: "Created in BioRender. Meyer, R. (2026) https://BioRender.com/4s9xc6n".

BioRender Content included in the Completed Graphic is not licensed for any commercial uses beyond use in a publication. For any commercial use of this figure, users may, if allowed, recreate it in BioRender under an Industry BioRender Plan.

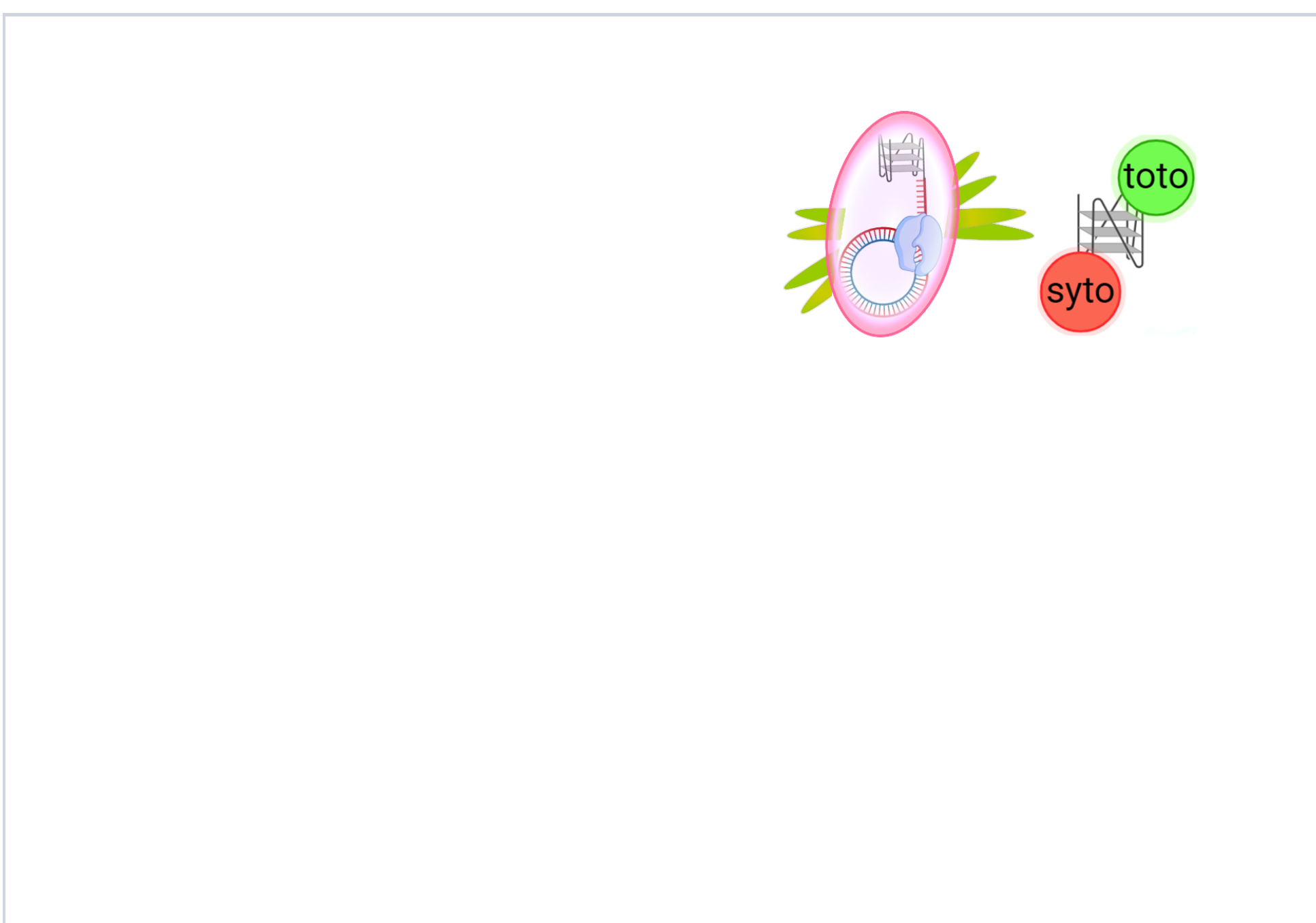

For any questions regarding this document, or other questions about publishing with BioRender, please refer to our BioRender Publication Guide, or contact BioRender Support at support@biorender.com.

49 Spadina Ave. Suite 200
Toronto ON M5V 2J1 Canada
www.biorender.com

Confirmation of Publication and Licensing Rights

August 9th, 2026

Subscription Type: *Institution - Academic*
Agreement number: *LX2A27K5JL*
Publisher Name: *Angewandte chemie*

Citation to Use: *Created in BioRender. Meyer, R. (2026) https://BioRender.com/d8ulv65*

To whom this may concern,

This document is to confirm that Rikke Meyer has been granted a license to use the BioRender Content, including icons, templates, and other original artwork, appearing in the attached Completed Graphic pursuant to BioRender's Academic License Terms. This license permits BioRender Content to be sublicensed for use in publications (journals, textbooks, websites, etc.).

All rights and ownership of BioRender Content are reserved by BioRender. All Completed Graphics must be accompanied by the following citation: "Created in BioRender. Meyer, R. (2026) https://BioRender.com/d8ulv65".

BioRender Content included in the Completed Graphic is not licensed for any commercial uses beyond use in a publication. For any commercial use of this figure, users may, if allowed, recreate it in BioRender under an Industry BioRender Plan.

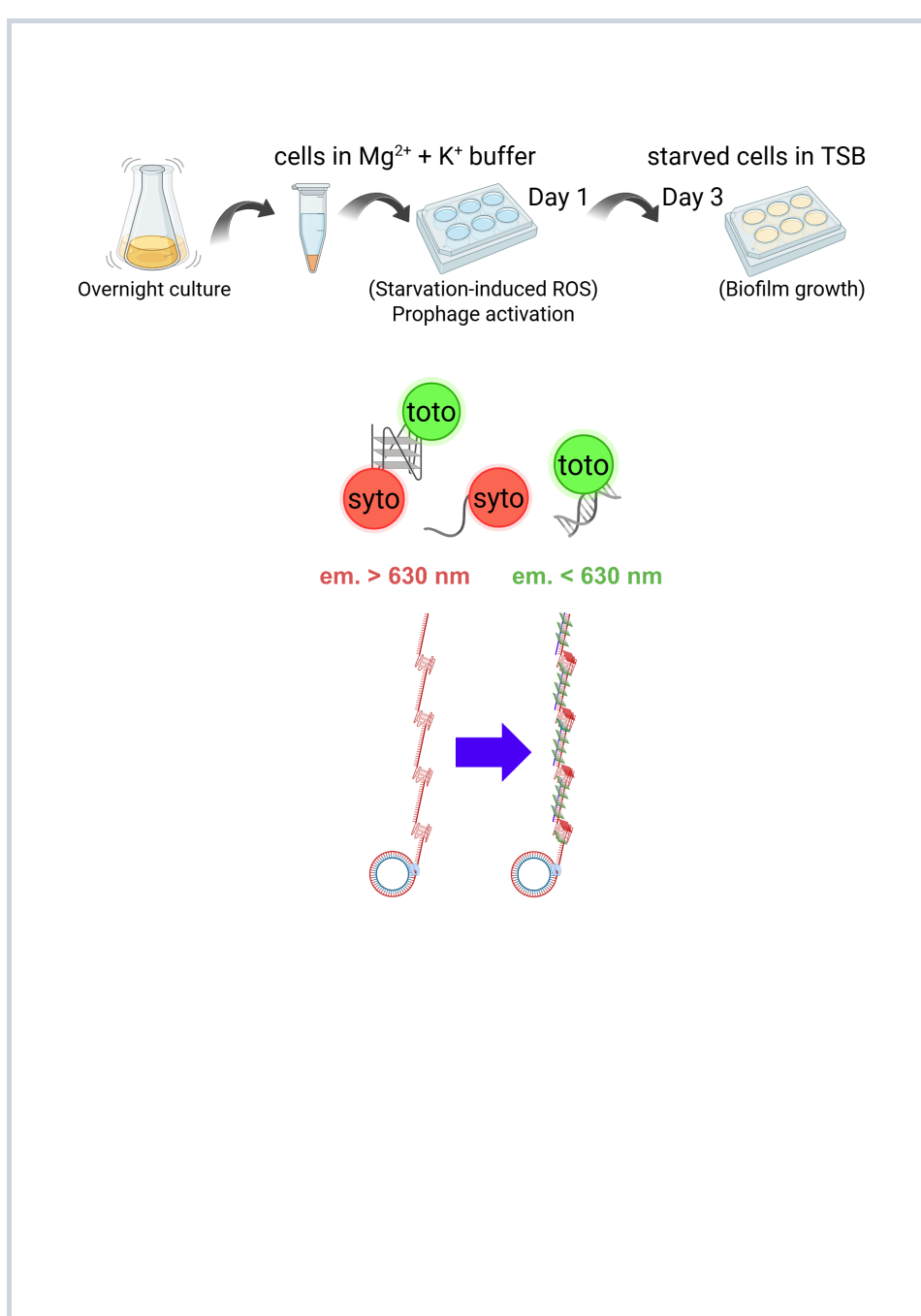

For any questions regarding this document, or other questions about publishing with BioRender, please refer to our BioRender Publication Guide, or contact BioRender Support at support@biorender.com.

49 Spadina Ave. Suite 200
Toronto ON M5V 2J1 Canada
www.biorender.com

Confirmation of Publication and Licensing Rights

August 9th, 2026

Subscription Type: *Institution - Academic*
Agreement number: *BR2A27JR2S*
Publisher Name: *Angewandte chemie*

Citation to Use: *Created in BioRender. Meyer, R. (2026) https://BioRender.com/tiroocc*

To whom this may concern,

This document is to confirm that Rikke Meyer has been granted a license to use the BioRender Content, including icons, templates, and other original artwork, appearing in the attached Completed Graphic pursuant to BioRender's Academic License Terms. This license permits BioRender Content to be sublicensed for use in publications (journals, textbooks, websites, etc.).

All rights and ownership of BioRender Content are reserved by BioRender. All Completed Graphics must be accompanied by the following citation: "Created in BioRender. Meyer, R. (2026) https://BioRender.com/tiroocc".

BioRender Content included in the Completed Graphic is not licensed for any commercial uses beyond use in a publication. For any commercial use of this figure, users may, if allowed, recreate it in BioRender under an Industry BioRender Plan.

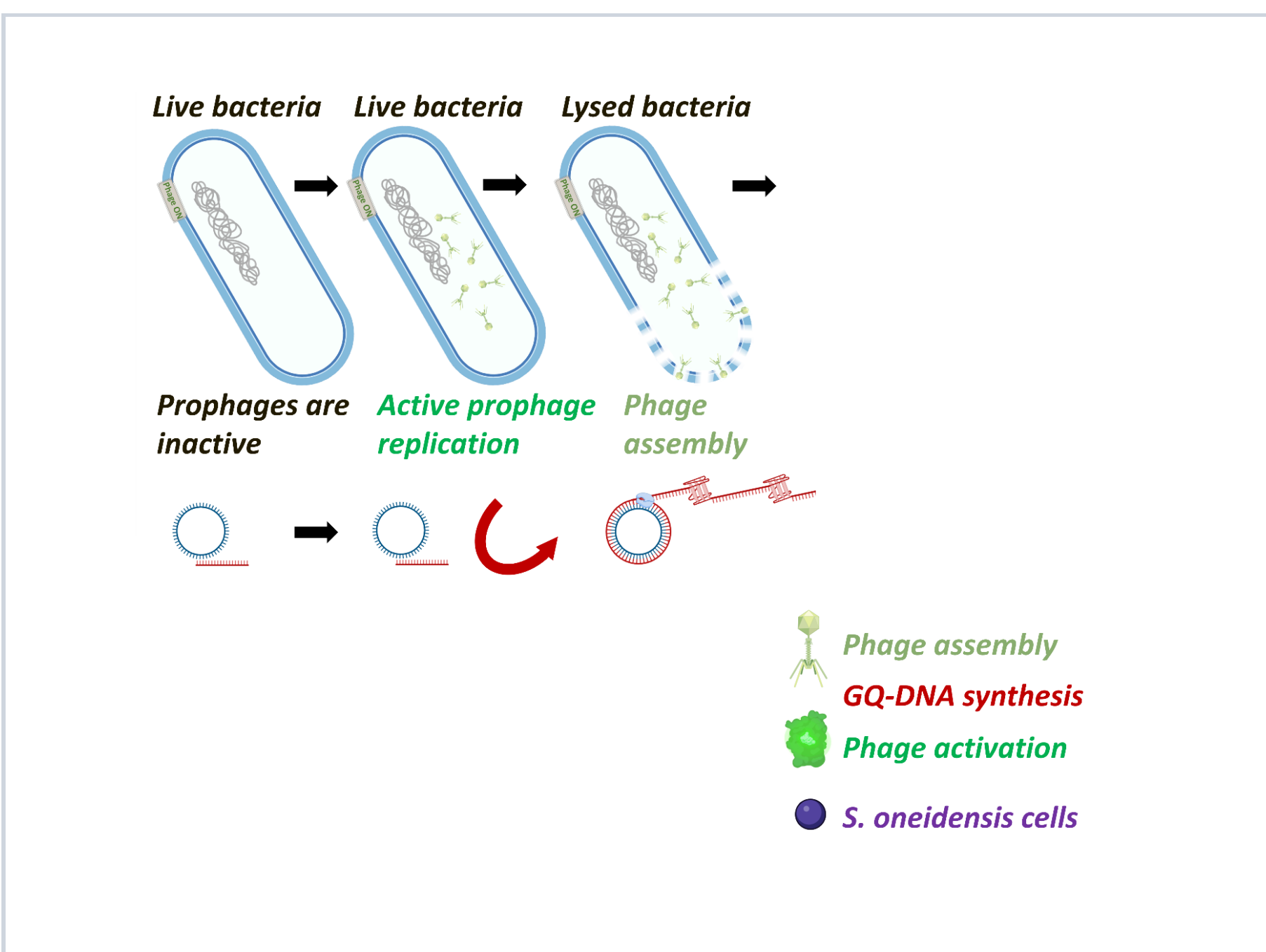

For any questions regarding this document, or other questions about publishing with BioRender, please refer to our BioRender Publication Guide, or contact BioRender Support at support@biorender.com.
